# Blockade of interferon signaling decreases gut barrier integrity and promotes severe West Nile virus disease

**DOI:** 10.1038/s41467-023-41600-3

**Published:** 2023-09-25

**Authors:** Shih-Ching Lin, Fang R. Zhao, Hana Janova, Adrian Gervais, Summer Rucknagel, Kristy O. Murray, Jean-Laurent Casanova, Michael S. Diamond

**Affiliations:** 1 Department of Medicine, Washington University School of Medicine, St. Louis, MO 63110 USA; 2 Laboratory of Human Genetics of Infectious Diseases, Necker Branch, Institut National de la Santé et de la Recherche Médicale (INSERM) U1163, Necker Hospital for Sick Children, Paris, EU 75015 France; 3 Paris Cité University, Imagine Institute, Paris, EU 75015 France; 4 Gnotobiotic Research, Education, and Transgenic Center, Washington University School of Medicine, St. Louis, MO 63110 USA; 5 Department of Pediatrics, Section of Pediatric Tropical Medicine, William T. Shearer Center for Human Immunobiology, Baylor College of Medicine and Texas Children’s Hospital, Houston, TX 77030 USA; 6 St. Giles Laboratory of Human Genetics of Infectious Diseases, Rockefeller Branch, The Rockefeller University, New York, NY 10065 USA; 7 Howard Hughes Medical Institute, New York, NY 10065 USA; 8 Department of Paediatrics, Necker Hospital for Sick Children, Paris, EU 75015 France; 9 Department of Pathology & Immunology, Washington University School of Medicine, St. Louis, MO 63110 USA; 10 Department of Molecular Microbiology, Washington University School of Medicine, St. Louis, MO 63110 USA; 11 Andrew M. and Jane M. Bursky the Center for Human Immunology and Immunotherapy Programs, Washington University School of Medicine, St. Louis, MO 63110 USA; 12 Center for Vaccines and Immunity to Microbial Pathogens, Washington University School of Medicine, St. Louis, MO 63110 USA

**Keywords:** West nile virus, Innate immunity

## Abstract

The determinants of severe disease caused by West Nile virus (WNV) and why only ~1% of individuals progress to encephalitis remain poorly understood. Here, we use human and mouse enteroids, and a mouse model of pathogenesis, to explore the capacity of WNV to directly infect gastrointestinal (GI) tract cells and contribute to disease severity. At baseline, WNV poorly infects human and mouse enteroid cultures and enterocytes in mice. However, when STAT1 or type I interferon (IFN) responses are absent, GI tract cells become infected, and this is associated with augmented GI tract and blood-brain barrier (BBB) permeability, accumulation of gut-derived molecules in the brain, and more severe WNV disease. The increased gut permeability requires TNF-α signaling, and is absent in WNV-infected IFN-deficient germ-free mice. To link these findings to human disease, we measured auto-antibodies against type I IFNs in serum from WNV-infected human cohorts. A greater frequency of auto- and neutralizing antibodies against IFN-α2 or IFN-ω is present in patients with severe WNV infection, whereas virtually no asymptomatic WNV-infected subjects have such antibodies (odds ratio 24 [95% confidence interval: 3.0 − 192.5; *P* = 0.003]). Overall, our experiments establish that blockade of type I IFN signaling extends WNV tropism to enterocytes, which correlates with increased gut and BBB permeability, and more severe disease.

## Abstract (web summary)

In this study, Lin and Zhao *et al*. show that type I IFN autoantibodies promote West Nile virus infection in enterocytes, which correlates with encephalitis disease risk in humans; while in mice, type I IFN signaling defects associate with gastrointestinal tract and blood-brain barrier permeability and disease severity.

## Introduction

West Nile virus (WNV) is a positive-stranded RNA flavivirus in the *Flaviviridae* family that is transmitted by mosquitoes and is related to other clinically relevant viruses, including dengue (DENV), Zika (ZIKV), Japanese encephalitis (JEV), yellow fever (YFV), and tick-borne encephalitis (TBEV) viruses. WNV infection of neurons in the central nervous system (CNS) can cause injury in the cerebral cortex, brain stem, and spinal cord^1,2^, and lead to severe disease including meningitis, encephalitis, and death in humans, horses, and other vertebrate animals^3^. The majority (approximately 80%) of WNV infections in humans are asymptomatic. Of the 20% of infections that cause symptoms, most individuals experience self-limited febrile illnesses with only 1% of infected individuals developing severe neuroinvasive disease^4,5^. While older age is known to be the biggest epidemiologic risk factor^6,7^, the immunologic and genetic determinants of severe WNV disease in this population are less well understood.

In several animal species, WNV also infects the gastrointestinal (GI) tract, resulting in immune cell infiltration, villus blunting, enterocyte necrosis, and intestinal dysmotility^8–10^. WNV infection of the GI tract occurs through hematogenous spread^10^, as the virus is inactivated by the acidic milieu of the stomach^11^. Reports of WNV infection in humans describe symptoms of GI tract infection or inflammation (*e.g*., vomiting, diarrhea, and/or abdominal pain)^12–14^. Although intestinal tissues from rodents, birds, and human patients show evidence of WNV RNA and antigen^8,10,15–18^, the tropism of WNV in the GI tract, apart from infection of neurons and its effects on pathogenesis, remains poorly characterized.

The type I interferon (IFN) signaling pathway is a first line of defense against many viral infections, including WNV^19^. IFNs induce expression of hundreds of IFN-stimulated genes (ISGs), a subset of which inhibit different steps in the infection cycle of viruses^20,21^. WNV infection and pathogenesis are restricted by pre-treatment of cells^22,23^ or animals^24^ with type I IFNs. Moreover, mice lacking type I IFNs or their shared IFNAR1/IFNAR2 receptor are vulnerable to subcutaneous inoculation of WNV^25,26^ and develop encephalits and lethal infection at high frequency^23^.

In humans, type I IFN antiviral responses can be compromised by rare genetic deficiencies in signaling and adaptor molecules (*e.g*., *TLR3, TLR7, IRF7, IFNAR1, IFNAR2*)^27,28^ or through the production of auto-antibodies (Abs) against type I IFNs^29,30^. In several cohorts of patients hospitalized with COVID-19 or influenza, the relative risk of severe disease and death was much greater in individuals with neutralizing Abs against type I IFN^31–37^. Related to this, auto-Abs to type I IFNs were associated with adverse reactions and outcomes after immunization with the live-attenuated vaccine against yellow fever, a flavivirus related to WNV^33^.

Here, we explored the dynamics of WNV infection of enterocytes and the GI tract by evaluating the effect of host IFN restriction on viral replication using human and mouse intestinal enteroids, and an established subcutaneous infection model in C57BL/6 J mice. Human and mouse intestinal organoid cultures have been used as models to study virus-host interaction in the GI tract^38–42^. Intestinal enteroids can be differentiated to multiple cell types including enterocytes, enteroendocrine cells, goblet cells, and Paneth cells^43^. When STAT1 signaling responses were absent, WNV more efficiently infected enteroids in culture. In vivo, greater enterocyte infection by WNV was observed after subcutaneous virus inoculation in animals with deficiencies in STAT1 or type I IFN signaling function. This pattern of infection was associated with enhanced disease and increased permeability of small molecules across GI tract and blood-brain barriers (BBB).

To begin to define the relevance of these findings, we tested convalescent sera for anti-type I IFN auto-Abs in a human WNV infection cohort, which includes asymptomatic subjects or those hospitalized with meningitis/encephalitis and severe disease^44–46^. Notably, a substantive fraction of severe WNV cases had measurable auto-Abs and neutralizing Abs against IFN-α2 or IFN-ω, two circulating type I IFN subtypes, whereas almost none of the asymptomatic subjects had these Abs. Treatment of human intestinal enteroids with serum from subjects with anti-IFN auto-Abs resulted in enhanced viral infection. Collectively, our experiments establish that defects in type I IFN signaling can enable WNV infection of epithelial cells in the GI tract, and this correlates with more severe disease in both mice and humans.

## Results

### WNV replicates in mouse and human intestinal organoids and is restricted by type I IFN responses

**Fig. 1:**
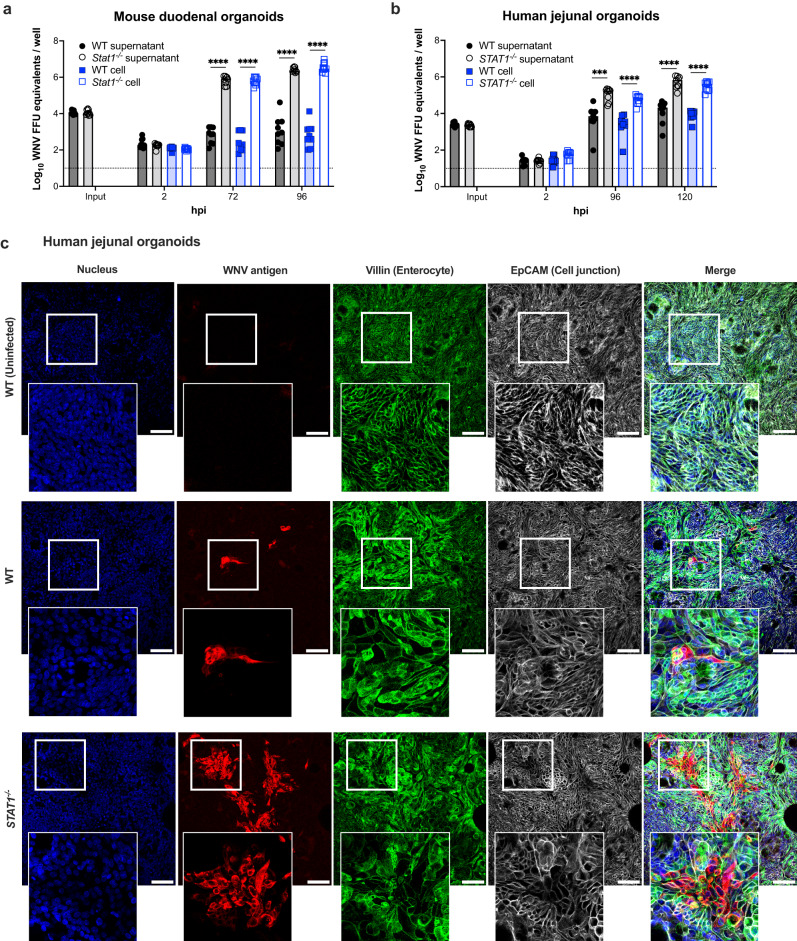
WNV infection of mouse and human intestinal enteroids is restricted by STAT1-dependent signaling. WT and STAT1-deficient mouse and human intestinal enteroids were inoculated with WNV (MOI of 0.5 for mouse and 0.1 for human enteroids) and cultured at 37 °C for the indicated time points. **a**, **b** Viral RNA extracted from supernatants and cell monolayers of mouse (**a**) or human (**b**) organoids was quantitated by RT-qPCR. Bars illustrate geometric means, and dotted lines show limits of detection (LOD). Each data point represents an individual well (*n* = 9 per group from 3 independent experiments). Statistical analysis: two-tailed Mann-Whitney test: ****P < 0.0001 (**a**); ***P = 0.0002, ****P < 0.0001 (**b**). **c** Human organoids cultured under air-liquid interface conditions were inoculated with WNV (MOI of 10) for 72 h, then fixed and stained with antibodies against WNV antigen (red), villin (green), and EpCAM (white), and counterstained for the nuclei (Hoechst 33258, blue). Scale bar, 100 μm. Data are representative of 3 experiments.

To begin to determine the permissivity of WNV infection for non-neuronal cells in the GI tract, we assessed whether WNV New York-1999 strain (herein, WNV) could productively infect mouse and human enteroids derived from healthy donors. Since *STAT1* is a key transcription factor downstream of IFN signaling that induces antiviral ISG expression and can be a determinant of cell and species tropism for viruses^47^, we first compared WNV infection in small intestinal (duodenum) cultures from wild-type and *Stat1*^-/-^ mice. All wild-type enteroid cultures showed limited viral replication at a multiplicity of infection (MOI) of 0.5, with less than 100-fold increases in viral RNA at 96 hours post-infection (hpi). In contrast, *Stat1*^-/-^ mouse enteroids were more permissive to WNV infection (Fig. 1a). For human enteroids, we inoculated wild-type (J2) and congenic *STAT1*^-/-^ (generated by CRISPR/Cas9 gene editing) jejunal enteroids with WNV. Again, limited infection was observed in wild-type enteroids, but substantially greater viral RNA levels (~ 22.4-fold increase at 96 hpi) were measured in the supernatants of *STAT1*^-/-^ enteroids (Fig. 1b). To identify the targets of WNV infection in the culture, we stained enteroid monolayers with Abs against WNV and different cell markers, and found that WNV infects villin- and EpCAM-expressing epithelial cells in *STAT1*-deficient monolayers, compared to limited infection in wild-type enteroids (Fig. 1c). Thus, STAT1-dependent signaling restricted WNV infection in vitro in both mouse and human intestinal epithelial-lineage cells.

### WNV infection in the GI tract is restricted by IFN responses

**Fig. 2:**
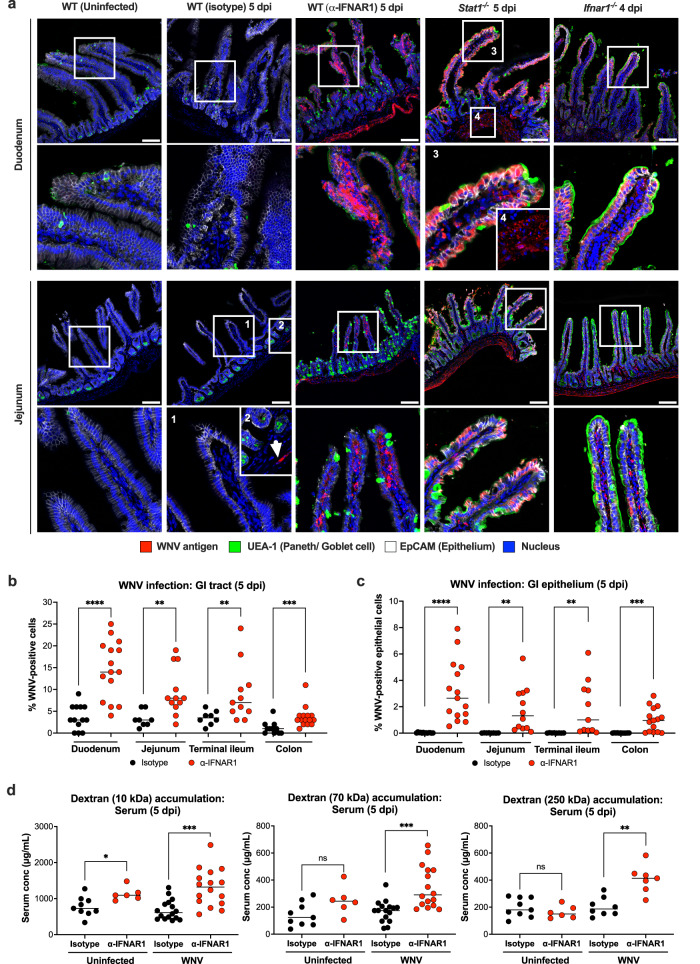
WNV infection in the GI tract is restricted by type I IFN responses. *Stat1*^*-/-*^ and *Ifnar1*^*-/-*^ mice, as well as WT mice treated with either isotype control or blocking IFNAR1 antibodies, were inoculated subcutaneously in the footpad with 10^2^ FFU of WNV. **a** Immunofluorescent confocal microscopy imaging of duodenal and jejunal cryosections of the GI tract at 4-5 dpi show WNV antigen (red), Paneth and goblet cells (UEA-1, green), EpCAM (white), and nuclei (Hoechst 33258, blue). Scale bar, 100 μm. Data are representative of 4 experiments; from left to right, *n* = 8, 13, 15, 8, and 8 mice per group. **b**, **c** Quantitation of WNV antigen positive cells in different regions along the GI tract was determined as a percentage of total Hoechst 33258-positive cells per field (**b**) or as a percentage of total EpCAM-positive epithelial cells per field (**c**). Lines indicate mean values. Data are from 4 experiments; from left to right, *n* = 13, 15, 8, 12, 8, 11, 11, and 15 per group. **d** Serum concentrations of 10, 70, and 250 kDa dextrans at 3 h after oral gavage in uninfected or WNV-infected (5 dpi) mice treated with anti-IFNAR1 or isotype control Ab. Data are from 3 experiments with lines indicating mean values; from left to right, *n* = 9, 6, 16, 16, 9, 6, 16, 16, 9, 6, 8, and 7 mice per group. Statistical analysis, from left to right: (**b**, **c**) two-tailed Mann-Whitney test: ****P < 0.0001, **P = 0.0013, **P = 0.0094, ***P = 0.0004 (**b**); ****P < 0.0001 (**c**); and (**d**) two-tailed Mann-Whitney test with Bonferroni correction: ns, not significant, ***P = 0.0006, ***P = 0.0003, **P = 0.0036.

To investigate the role of STAT1 and IFN signaling in modulating WNV tropism in the GI tract, we inoculated 9-10-week-old male wild-type, *Ifnar1*^-/-^, *Ifngr*^-/-^ (loss of IFN-γ signaling), *Ifnlr1*^-/-^ (loss of IFN-λ signaling), and *Stat1*^-/-^ mice via a subcutaneous route with 10^2^ focus-forming units (FFU) of WNV. We also examined the effects of an acquired deficiency of type I IFN signaling by treating wild-type mice with a blocking anti-IFNAR1 Ab prior to WNV inoculation. At 5 days post-infection (dpi), wild-type mice showed sparse enteric neuronal infection in the myenteric plexus without apparent epithelial cell infection in the GI tract (Fig. 2a). Similar results were observed in *Ifngr*^-/-^ and *Ifnlr1*^*-/-*^ mice, suggesting that type II and III IFN pathways do not have dominant roles in restricting WNV infection of GI tract epithelial cells (Supplementary Fig 1a). In comparison, both *Ifnar1*^-/-^ and *Stat1*^-/-^ mice showed extensive WNV antigen staining in the myenteric plexus, the lamina propria, and enterocyte layers, with greater infection in the duodenum than other intestinal segments (Fig. 2a and Supplementary Fig 1b); as the enterocyte infection phenotype appeared more prominent in *Stat1*^-/-^ than *Ifnar1*^-/-^ mice, signaling from IFN-λ, IFN-γ, or other cytokines upstream of STAT1 signaling might still contribute to control of WNV infection in the GI tract, particularly in the absence of type I IFN signaling. Consistent with these results, wild-type mice treated with a blocking anti-IFNAR1 antibody also showed extensive WNV infection throughout the GI tract at 5 dpi, with antigen staining that was similar in distribution to that seen in *Ifnar1*^-/-^ mice (Fig. 2a,b).

### WNV infection alters barrier permeability in type I IFN signaling-deficient mice

Given that WNV can infect the intestinal epithelium (enterocytes) in mice with deficient type I IFN signaling (Fig. 2c), we investigated the functional effects on barrier integrity. To evaluate GI tract permeability at 5 dpi, we administered fluorescently-conjugated dextrans of different molecular weights (Cascade Blue [10 kDa], Tetramethylrhodamine [70 kDa], and FITC [250 kDa]) to WNV-infected mice by oral gavage and measured translocation into circulation. Compared to uninfected or isotype control Ab-treated WNV infected animals, WNV-infected mice that received anti-IFNAR1 antibody accumulated higher levels of all three dextrans in their sera (Fig. 2d), indicating that deficient type I IFN signaling and associated WNV infection of the GI tract correlates with increased intestinal barrier permeability. However, culture of blood did not yield any bacterial growth (Supplementary Table 1), suggesting that gut barrier integrity was compromised principally for smaller molecules such as constituents of bacteria and dietary products.

**Fig. 3:**
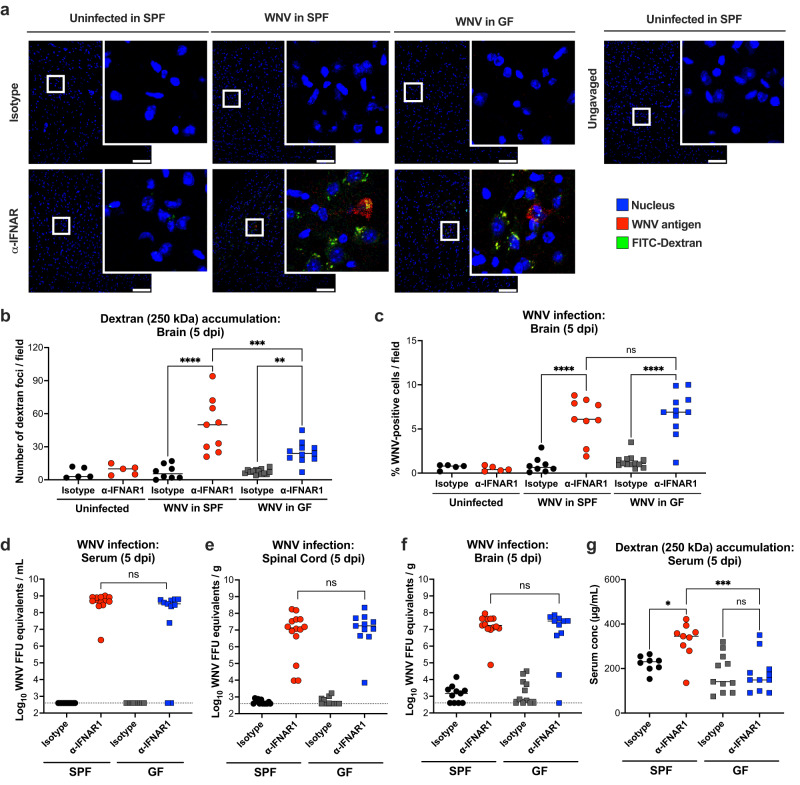
Type I IFN signaling-deficiency alters accumulation of GI tract-derived molecules in the brains of WNV infected mice. **a**–**c** Uninfected or WNV-infected (5 dpi) specific pathogen free (SPF) or germ-free (GF) mice treated with either isotype control or blocking IFNAR1 antibodies were administered 250 kDa FITC-dextran by oral gavage. Confocal microscopy imaging of brain sections shows WNV-antigen (red), dextran (green), and nuclei (Hoechst 33258, blue). Scale bars, 100 μm, and high-power insets are shown from the boxed regions (**a**). Accumulation of translocated dextran was quantitated as the number of foci per field (**b**). WNV antigen staining was quantitated as a percentage of total Hoechst 33258-positive cells per field (**c**). Data are from 3 experiments with lines indicating mean values; from left to right, *n* = 5, 5, 8, 9, 13, and 11 (**b-c**). **d**–**f** WNV RNA levels in serum (**d**), spinal cord (**e**), and brain (**f**) were determined by RT-qPCR. Data are from 3 experiments with solid lines indicating geometric mean values and dotted lines showing limits of detection (LOD); from left to right, *n* = 11, 13, 11, and 11 mice per group (**d** and **f**); *n* = 12, 14, 11 and 11 mice per group (**e**). **g** Accumulation of orally-gavaged 250 kDa FITC-dextran in the serum of WNV-infected SPF and GF mice at 5 dpi that were treated with the indicated antibodies. Lines indicate mean values. Data are from 3 experiments; from left to right, *n* = 8, 9, 11, and 11 mice per group. Statistical analysis, from left to right: (**b**–**c,** and **g**) one-way ANOVA with Šídák’s post-test: ****P < 0.0001, ***P = 0.0005, **P = 0.0059 (**b**); ****P < 0.0001, ns, not significant, ****P < 0.0001 (**c**); *P = 0.0249, ***P = 0.0004, ns, not significant (**g**) and (**d**–**f**) two-tailed Mann-Whitney test: ns, not significant.

We next evaluated whether the enhanced GI tract permeability led to accumulation of gut constituents in the brains of anti-IFNAR1-treated mice. At 5 dpi, WNV-infected mice that were treated with anti-IFNAR1 antibody showed evidence of fluorescently-labelled dextran in the brain (Fig. 3a-b) that was administered by oral gavage. Uninfected mice or WNV-infected mice with intact type I IFN responses did not show this accumulation. These findings correlated with WNV infection, with anti-IFNAR1 antibody-treated mice showing higher levels of WNV antigen in the GI tract (Fig. 2a-c) and brain (Fig. 3c), and viral RNA in the serum, spinal cord, and brain compared to animals treated with the isotype control antibody (Fig. 3d-f). These in vivo studies suggest that the GI tract barrier is impaired after WNV infection when type I IFN responses are compromised, and this correlates with enhanced infection in these tissues. However, we did not observe an increase in gut permeability to the 250 kDa FITC-dextran that we measured after anti-IFNAR1 treatment at 3 dpi (Supplementary Fig 2b), even though viral RNA was present in the serum, liver, and brain (Supplementary Fig 2c-e).

We separately assessed the effect of anti-IFNAR1 treatment on BBB permeability after WNV infection by intravenously administering fluorescently conjugated dextrans to bypass effects in the GI tract. At 5 dpi, anti-IFNAR1-treated mice showed evidence of increased BBB permeability and accumulation of dextrans in the perivascular space and parenchyma of several regions of the brain including the olfactory bulb, brainstem, and cerebellum (Supplementary Fig 3a-f). This result is consistent with prior data showing that type I IFNs stabilize the BBB tight junctions after injection of pathogen-assocoated molecular patterns or infection by WNV^48^.

### GI tract permeability and disease severity is diminished in germ-free mice

We next evaluated whether GI tract barrier permeability changes associated with type I IFN blockade and WNV infection may be linked to the presence of a gut microbiota, since commensal bacterial components can modulate cellular junctions and barrier integrity^49,50^. We treated 9-10-week-old germ-free (GF) C57BL/6 J mice with isotype control or anti-IFNAR1 antibody, followed by subcutaneous inoculation with 10^2^ FFU of sterile-filtered WNV. At 3 or 5 dpi, we orally gavaged GF animals with 250 kDa FITC-conjugated dextran. At 3 dpi, no differences in GI tract permeability were observed with anti-IFNAR1 treatment of specific pathogen-free (SPF, conventional microbiota) and GF mice (Supplementary Fig 2a), although moderately lower levels of viral RNA were observed in the liver (4-fold, *p* < 0.01) and brain (4-fold, *p* < 0.05) but not serum of GF mice (Supplementary Fig 2c-e). At 5 dpi, and in contrast to that seen in anti-IFNAR1 antibody-treated SPF mice, GF mice did not show enhanced GI tract pemeability when type I IFN signaling was blocked (Fig. 3g) even though substantial WNV infection was present in enterocytes from all segments of the GI tract (Supplementary Fig 4a-c). Similarly, GF mice treated with anti-IFNAR1 antibody showed reduced BBB permeability compared to similarly treated SPF mice at 5 dpi (Supplementary Fig 3). Additionally, although anti-IFNAR1 antibody-treated GF mice exhibited higher levels of orally gavaged FITC-dextran accumulation in the brain compared to isotype control antibody-treated GF mice, the level was lower than in conventionally-housed SPF animals (Fig. 3a-b). Nonetheless, similar levels of WNV infection were detected in the serum, brain, and spinal cord of anti-IFNAR1-treated GF animals by antigen staining (Fig. 3a and c) or RT-qPCR (Fig. 3d-f) at 5 dpi. These results suggest that while certain components of the host microbiota may affect GI tract and BBB permeability, and accumulation in the brain of circulating molecules or proteins, WNV may use other mechanisms [*e.g*., Trojan horse effect of WNV-infected blood cells, direct infection of endothelial cells, or transcytosis of endothelial cells^51–53^] to invade the CNS.

As prior studies also implicated TNF-α receptor signaling in WNV-induced changes in BBB permeability^54,55^, we determined its importance for regulating GI tract and BBB permeability in the context of WNV infection and type I IFN signaling blockade. In the setting of type I IFN signaling blockade, anti-TNF-α antibody treatment resulted in decreased GI tract permeability to 250 kDa dextrans (Supplementary Fig 5a), reduced BBB permeability (Supplementary Fig 3**)**, and reduced dextran translocation from the GI tract to the brain (Supplementary Fig 5b-c) even though substantial WNV infection in the GI tract was observed by antigen staining (Supplementary Fig 6a-b), indicating that the reductions of gut and BBB permeability were related to abrogated TNF-α signaling instead of altered WNV infection. Indeed, WNV infection in the brain was similar in anti-IFNAR1/anti-TNF-α and anti-IFNAR1/isotype control-treated mice when assessed by antigen staining, although small reductions were detected in the serum (2-fold, *p* = 0.049), but not in the brain, of anti-IFNAR1/anti-TNF-α treated mice by RT-qPCR (Supplementary Fig 5b, d-f). These data suggest that the enhancement of GI tract permeability seen after WNV infection occurs in part through TNF-α signaling, although the impact on viral load in the brain was small, as we observed in GF mice (Fig. 3c and f).

**Fig. 4:**
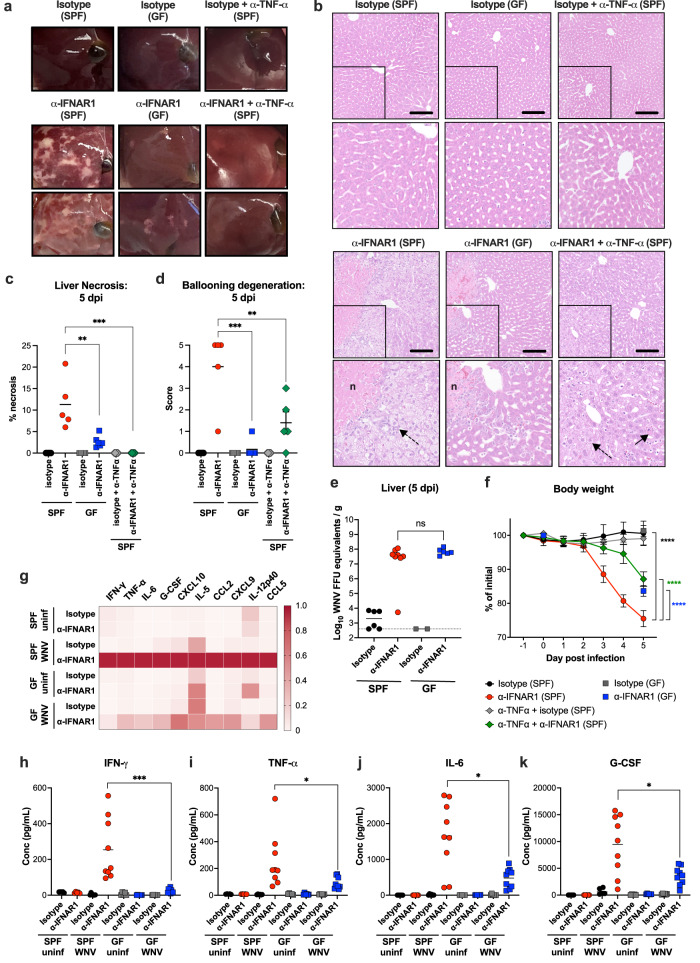
Differences in inflammation and hepatic injury in WNV-infected anti-IFNAR1-treated SPF and GF mice. **a** Representative gross pathology images of the liver of WNV infected (5 dpi) SPF or GF mice treated with the indicated antibodies (3 experiments, *n* = 6–8 mice per group). Pale white areas in the parenchyma represent regions of necrosis. **b**–**d** Hepatic injury. Hematoxylin and eosin staining of liver sections from isotype control or anti-IFNAR1 treated SPF or GF mice at 5 dpi. Some of the SPF animals also received anti-TNF-α antibody beginning one day before subcutaneous WNV inoculation. Boxed insets are shown immediately below at higher magnification. The letter **n** denotes necrosis, dotted arrows show ballooning hepatocytes, and the solid arrow shows an acidophilic body with karyorrhexis (apoptotic body). Scale bars indicate 100 μm (**b**). Quantitation of liver injury as determined by the extent of necrosis (**c**) and ballooning degeneration (**d**). Scoring index is described in the Methods. Lines indicate mean values; from left to right, results are from *n* = 5, 5, 5, 6, 5, and 5 mice per group. **e** WNV RNA levels in liver at 5 dpi were determined by RT-qPCR. Results are from 2 experiments with solid lines indicating mean values and dotted line indicating LOD; from left to right, *n* = 6, 8, 2, and 6 mice per group. **f** SPF and GF mice receiving different treatments were measured for body weight daily or at 0 and 5 dpi (for GF mice only). Data from 2 experiments and presented as the mean ± SEM; from top to bottom, *n* = 7, 7, 6, 6, 7, and 9 mice per group. **g**–**k** Cytokine levels in serum. GF and SPF mice were treated with isotype control or anti-IFNAR1 antibody. Some of the animals were then infected with WNV via subcutaneous inoculation. At 5 dpi, serum was harvested, and cytokines were measured using a multiplexed assay (see Methods). A heat map of cytokine levels, normalized to the highest value for each cytokine, is shown (**g**). Absolute serum levels of IFN-γ (**h**), TNF-α (**i**), IL-6 (**j**), and G-CSF (**k**) are shown. Data are from 2 experiments with lines indicating the mean values; from left to right, *n* = 5, 5, 6, 9, 5, 6, 6, and 9 mice per treatment group. Statistical analysis: (**c**, **d**) one-way ANOVA with Dunnett’s post-test: **P = 0.0019, ***P = 0.0003 (**c**); **P = 0.008, ***P = 0.0003 (**d**); (**e**) two-tailed Mann-Whitney test: ns, not significant; (**f**) two-way ANOVA with Dunnett’s post-test: ****P < 0.0001; (**h**–**k**) two-tailed Mann-Whitney test with Bonferroni correction: ***P = 0.003 (**h**); *P = 0.047 (**i**); *P = 0.019 (**j**); *P = 0.048 (**k**).

Because blood from the GI tract drains into the liver via the portal vein, we evaluated whether hepatic injury differentially occurred in SPF and GF mice treated with anti-IFNAR1 antibodies in the context of WNV infection. Whereas WNV-infected SPF mice treated with anti-IFNAR1 antibody demonstrated evidence of extensive liver necrosis at 5 dpi, similarly treated GF mice showed much less macroscopic (Fig. 4a) and microscopic (Fig. 4b-d) liver pathology, despite having no differences in WNV RNA levels in the liver at this time point (Fig. 4e). These results suggest that WNV infection of gut-associated cells and increased translocation of gut constituents into circulation after type I IFN signaling blockade in SPF mice can promote tissue inflammation and damage. The necrotic liver injury was not due to bacterial infection, as culture of liver homogenates from WNV-infected anti-IFNAR1-treated SPF mice did not yield bacterial growth (Supplementary Table 1). To determine the clinical consequences of enhanced GI tract infection and permeability, we measured changes in body weight of mice after WNV infection and anti-IFNAR1 treatment. Notably, GF mice treated with anti-IFNAR1 antibodies exhibited less weight loss after WNV infection than similarly-treated SPF mice (Fig. 4f). Moreover, anti-IFNAR1/anti-TNF-α-treated SPF mice also showed less weight loss than SPF mice that received anti-IFNAR1 but not anti-TNF-α antibody (Fig. 4d), indicating the TNF-α signaling is critical for WNV pathogenesis in this setting.

Given the findings with anti-TNFα antibody, we broadly measured serum cytokine levels in SPF and GF mice in the context of type I IFN signaling blockade at 5 dpi. Anti-IFNAR1-treated WNV-infected SPF mice accumulated substantially higher levels of pro-inflammatory cytokines and chemokines (IFN-γ, TNF-α, IL-6, G-CSF, CXCL10, IL-5, CCL2, CXCL9, IL-12p40, and CCL5) than WNV-infected SPF mice treated with an isotype-control antibody or uninfected mice treated with either anti-IFNAR1 or isotype control antibody (Fig. 4g-k and Supplementary Fig 7). However, the accumulation of pro-inflammatory mediators in serum after WNV infection and anti-IFNAR1 treatment was potentially microbiota-dependent, since GF mice showed lower levels of several key cytokines (IFN-γ, TNF-α, IL-6, G-CSF) (Fig. 4g-k). Because pro-inflammatory cytokines can impact microglia or macrophage activation and neuronal function in the brain^56–58^, we assessed the effects of anti-IFNAR1 treatment in SPF and GF mice. Compared with the brains of uninfected SPF mice or WNV-infected anti-IFNAR1-treated GF mice, anti-IFNAR1-treated SPF mice at 5 dpi showed more prominent changes in morphology and greater numbers of Iba1^+^ cells (Supplementary Fig 8a-c), that are characteristic of activated microglia and/or increased infiltration of monocyte-derived macrophages^59^.

### Auto-Abs against type I IFN in human patients with severe WNV infection

Recent studies have suggested that pathological responses to live-attenuated yellow fever vaccine^33^, severe influenza^35^ and SARS-CoV-2 infections^31,32,35,36^, occur more frequently in older humans with auto-Abs to type I IFNs^31–33,35,36^. Given that severe WNV infection in the CNS occurs in the elderly^60^, we hypothesized that part of the enhanced risk might be due to an acquired deficiency of type I IFN signaling, which could impact infection and barrier permeability. To test this idea, we acquired convalescent serum from a cohort of asymptomatic WNV-infected subjects who were identified as viremic through blood bank donations (*n* = 19) and a separate cohort of hospitalized subjects with severe WNV neuroinvasive disease, all diagnosed with encephalitis (*n* = 56; Houston West Nile Cohort, 2002-2018)^44–46,61^ (Table 1). There were no significant differences between the demographic characteristics of patients in the asymptomatic and neuroinvasive WNV groups, including age and sex.

**Table 1:** Demographics and clinical data of WNV-infected subjects

	Asymptomatic WNV *N* = 19	Neuroinvasive WNV *N* = 56
Age, years	57 (23)	59.5 (21)
Male sex	84%	75%
Ethnicity/Race		
Caucasian	94.7%	76.8%
African American	5.3%	8.9%
Hispanic	0%	10.7%
Asian or Pacific Islander	0%	3.6%
Neurological symptoms	–	
Fever		68%
Altered mental status		61%
Tremors		25%
Dysequilibrium		20%
Blurred vision		18%
Paralysis		18%
Seizures		16%
Memory loss		14%
Language		9%
Loss of consciousness		7%
Coma		5%
Stroke		2%
Other signs or symptoms	–	
Gastrointestinal		48%
Rash		25%
Musculoskeletal		23%
Cardiopulmonary		7%
Genitourinary		7%

Subjects presenting with multiple clinical symptoms are listed under each manifested symptom. Values expressed as % or median (interquartile range). WNV, West Nile virus.

**Fig. 5:**
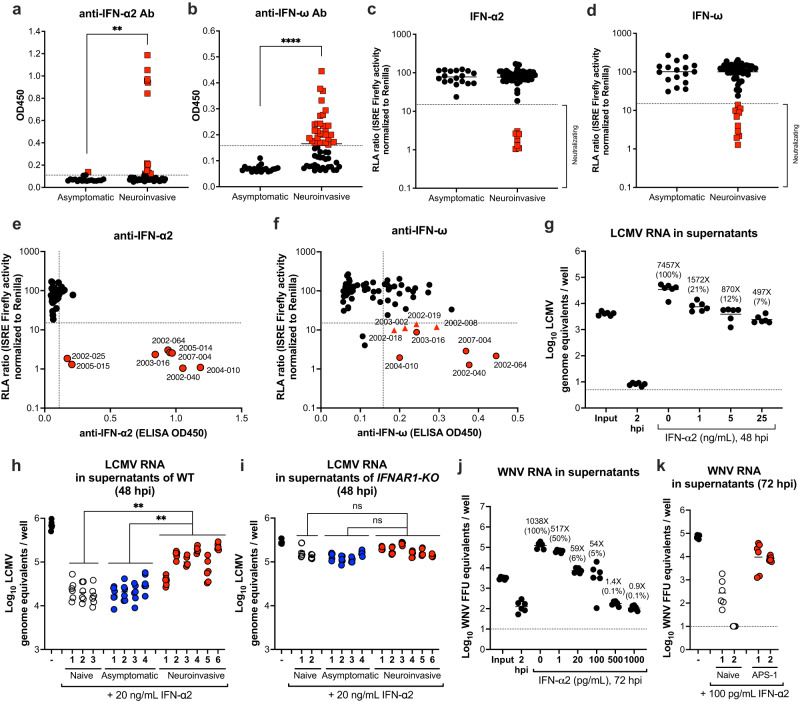
Auto-Abs against type I IFN in human patients with WNV infection. **a**, **b** The optical density (O.D. 450 nm) values of anti-IFN-α2 (**a**) and anti-IFN-ω (**b**) auto-Abs in asymptomatic (*n* = 19) and neuroinvasive (*n* = 56) WNV cohorts. Samples with positive signals are colored red. **c**, **d** Luciferase reporter assay measuring serum neutralization of exogenous IFN-α2 (**c**) and IFN-ω (**d**) from subjects who experienced asymptomatic (*n* = 18) or neuroinvasive WNV (*n* = 56) infection. An RLA ratio lower than 15% was defined as neutralization (red colored samples), as described previously^32^. **e**, **f** Correlation of ELISA and Luciferase reporter assay results for detecting auto-Abs against IFN-α2 (**e**) and IFN-ω (**f**) with neutralizing activity. In **e** and **f**, samples with red circles neutralize IFN-α2. In **f**, samples with red triangles neutralize IFN-ω only. Data are from 1 experiment performed in duplicate, and the mean values are shown. **g**–**i** Human intestinal enteroids in wild type (**g, h**) or *IFNAR1* KO backgrounds (**i**) were pretreated with 0, 1, 5 and 25 ng/mL of IFN-α2 for 8 h (**g**) or with 20 ng/mL IFN-α2 in the presence of 10% patient serum sample for 8 h (**h, i**). The treated cultures were inoculated with LCMV (MOI of 0.1) for 2 h. Supernatants were harvested at 2 (input) or 48 hpi and assayed for viral RNA by RT-qPCR. Fold differences were calculated by the increase of LCMV genome equivalents compared to 2 hpi. **j**, **k** Human enteroids were pretreated with 0, 1, 5 and 25 ng/mL of IFN-α2 for 8 h (**j**) or with 100 pg/mL of IFN-α2 in the presence of 10% sera (from naive wild-type or APS-1 donors) for 8 h (**k**). The treated cultures were inoculated with WNV (MOI of 0.1) for 2 h. Supernatants were harvested at 2 (input) or 72 hpi and assayed for viral RNA. Fold values were calculated by the increase of WNV FFU equivalents compared to the 2 hpi collection. Lines indicate geometric means and dotted lines show LOD. Each data point represents an individual well with *n* = 6 wells per group from 2 experiments. Color definitions: untreated (black), sera from naïve (uninfected) individuals (white), sera from asymptomic infections (blue), sera from neuroivasive infections (red) (**h**–**i**) or sera from APS-1 patients (red) (**k**). Statistical analysis: (**a**–**b**) two-tailed Mann–Whitney test: **P = 0.0047 (**a**); ****P < 0.0001 (**b**); (**h**–**i**) one-way ANOVA with Dunnett’s post-test, from left to right: **P = 0.002, **P = 0.0016 (**h**); ns, not significant (**i**).

We measured anti-IFN-α2 and IFN-ω auto-Abs by ELISA, and used sera from 5 healthy donors who had no known WNV exposures as negative controls to set cutoff values. Anti-IFN-α2 auto-Abs were more prevalent in sera from subjects with WNV meningitis or encephalitis (14 of 56, 25.0%) than asymptomatic individuals (1 of 19, 5.3%, Fig. 5a). Similar results were observed when we measured auto-Abs against IFN-ω (29 of 56, 51.8% with neuroinvasive infection and 0% in asymptomatic) (Fig. 5b). Although we observed a similar trend for anti-IFN-β auto-Abs in severe infection cases, our detection assay was not robust enough to be conclusive (Supplementary Fig 9). Based on these data, our case-control study revealed an odds ratio of 24 (95% confidence interval, 3.0–192.5; *P* = 0.003) for severe neuroinvasive WNV disease in association with the presence of auto-Abs against IFN-α2 and/or IFN-ω.

We corroborated these results using an established luciferase ISG reporter gene assay^32^ to evaluate the ability of patient sera to prevent induction of IFN signaling by reporter cells in response to exogenous type I IFN. Notably, 8 of 56 (14.3%) and 11 of 56 (19.6%) serum samples from subjects with severe neuroinvasive disease neutralized responses to recombinant IFN-α2 and IFN-ω, respectively (Fig. 5c-d). Additionally, 5 of the sera contained auto-Abs that neutralized both type I IFNs (Fig. 5e-f). All of the samples with neutralizing activity against IFNs were from subjects who experienced WNV infection and with severe neuroinvasive disease.

To link these results with our GI tract infection data, we evaluated whether sera from WNV patients with auto-IFN Abs could blunt antiviral type I IFN responses in human enteroids. In a first set of experiments, we tested for effects of sera on infection by an IFN-sensitive virus (lymphocytic choriomeningitis virus [LCMV]); we did not use WNV for these studies because the sera from convalescent WNV-infected subjects had neutralizing anti-WNV antibodies. LCMV infection of human enteroids was restricted by pretreatment of cultures with IFN-α2 (Fig. 5g). When IFN-α2 was pre-mixed with human sera from selected WNV subjects who experienced neuroinvasive disease and contained auto-Abs to type I IFN, LCMV replication was restored (Fig. 5h). As the restoration was not observed in *IFNAR1*^*-/-*^ human organoids with the same treatment (Fig. 5i), the increased of LCMV replication was due to the neutralization of IFN-α2 by human sera. Next, to test the effect of sera containing type I IFN auto-Abs on WNV infection of human enterocytes, we utilized sera from patients with autoimmune polyglandular syndrome type 1 (APS-1) that contain auto-Abs against type I IFNs but had never been infected with WNV (verified by prescreening ELISA). As expected, WNV infection was inhibited by IFN-α2 pretreatment (Fig. 5j), and the addition of sera from APS-1 patients restored WNV infection in human enteroid cultures (Fig. 5k). Thus, in the subset of WNV encephalitis human patients that develop high levels of auto-Abs to type I IFN, viral infection of enteric cells is likely greater, which could impact disease severity through effects on viral replication and barrier permeability.

## Discussion

In this study, we showed that human and mouse enteroids are largely resistant to WNV infection unless STAT1 signaling is absent due to genetic defects. Analysis of GI tract tissues from mice demonstrated that enterocytes, especially from the duodenum, can be targeted by WNV when blocking anti-IFNAR1 antibodies are administered or in the context of genetic deficiencies in STAT1 and IFNAR1 but not IFNLR1 or IFNGR. The lack of WNV infection of enterocytes in *Ifnlr1*^-/-^ mice was unexpected given that IFN-λ is an important antiviral restriction factor for other viruses that infect epithelial cells in the GI tract including norovirus and rotavirus^62–65^. Possibly, a deficiency of IFN-λ signaling (or IFN-γ signaling), by itself, is not sufficient to enable WNV to access enterocytes due to a lack of infection of underlying lamina propria cells. Unlike norovirus and rotavirus, which infect enterocytes from the gut lumen after oral ingestion, WNV infects the skin and spreads via the blood to the myenteric plexus and lamina propria of GI tract, and likely infects enterocytes from the basolateral side. Indeed, we did not observe enterocyte infection by WNV after subcutaneous inoculation of Villin-Cre x *Stat1*^*fl/fl*^ mice, which lack STAT1 expression only in GI tract epithelial cells (Supplementary Fig 10a), likely because WNV did not productively infect the underlying stromal and/or immune cells (Supplementary Fig 10b). In contrast, a deficiency of type I IFN signaling in myeloid cells was previously shown to cause dysregulated cytokine responses, complement activation, sepsis, and organ damage, although intestinal infection was not evaluated in that study^66^.

In the setting of type I IFN signaling deficiencies, enhanced WNV infection in the lamina propria and enterocytes was associated with greater GI tract permeability to different sized dextrans, but not to bacteria. These dextrans accumulated at higher levels in peripheral circulation and in the brain. WNV infection in the setting of type I IFN signaling blockade also was associated with changes in BBB permeability, as reported previously^48^. Together, these effects resulted in more severe clinical disease. However, changes in GI tract permeabiliy and accumulation of dextrans in the brain were decreased in WNV-infected, anti-IFNAR1 antibody-treated GF mice and in anti-TNF-α antibody-treated SPF mice, suggesting that elements of the microbiota may be required for inducing pro-inflammatory signals and cytokines, particularly TNF-α, that modulate barrier permeability. These findings are consistent with the concept that changes in the microbiota composition can modulate functions in the brain via actions of pro-inflammatory cytokines^67–69^, and thus, modify neurological outcomes of virally-infected patients^69,70^.

Because deficiencies in IFN signaling were associated with WNV infection in enteroids and enterocytes in vivo, we hypothesized that severe neuroinvasive WNV disease might be associated with auto-Abs to type I IFNs. Indeed, in a cohort of 75 WNV-infected subjects from Texas, the frequency of auto-Abs to IFN-α2 and IFN-ω was greater in individuals with encephalitis than those with asymptomatic infection. Our results were recently corroborated by a larger cohort study, which analyzed 401 WNV-infected subjects from the European Union and the United States of America, and concluded that auto-Abs neutralizing type I IFNs are associated with approximately one-third of the cases of severe WNV infection requiring hospitalization^71^. Nonetheless, in humans, the precise cellular mechanism by which auto-Abs to IFN predispose to severe disease remains uncertain and could reflect increased burden of infection, altered cellular tropism of infection in multiple tissues, and/or changes in BBB permeability due to local loss of IFN signaling and antiviral immunity, as well as effects in the GI tract and at other sites^72^. Indeed, bacterial constituents (*e.g*., lipids, cell wall components, and nucleic acids) and type I IFN signaling both can directly regulate BBB integrity and function^48^.

The role of the gut microbiota in WNV pathogenesis can depend on the immune status of the host. In immunocompetent C57BL/6 mice, antibiotic-induced perturbations in the microbiota can negatively impact CD8^+^ T cell responses, which affect viral clearance and survival^73^; in this case, an intact and balanced microbiota had a protective role in instructing adaptive T cell responses against WNV infection. However, in the context of an innate immune deficiency (e.g. *Ifnar1*^*-/-*^ mice, anti-IFNAR1 blocking antibody treatment of mice, and by inference auto-antibodies to type I IFNs in humans), the microbiota appears to have a detrimental role because it is a source of PAMPs that exacerbate systemic inflammation in the setting of enhanced GI tract infection and loss of barrier integrity. Thus, in immunocompetent and immunodeficient animals, the microbiota can have distinct effects on innate and adaptive immune responses and clinical outcomes during virus infection.

### Limitations of the study

We acknowledge several limitations in our experiments: (a) for the organoid cultures, we did not address the effects of the microbiota, bioactive metabolites, or host immune cells, all of which could independently impact WNV infection, cell survival, and cell structural integrity; (b) we did not definitively determine the tropism of WNV in specific enterocyte subpopulations in enteroids or in vivo, which will require further immuohostochemical staining with additional cell-type specific markers or single cell RNA sequencing experiments; (c) while our staining experiments suggest that brain microglia and macrophages are differentially activated in WNV-infected anti-IFNAR1-treated SPF and GF mice, the mechanism was not defined. Experiments with antibodies that block the functions of the pro-inflammatory cytokines that were differentially expressed in serum of WNV-infected anti-IFNAR1-treated SPF and GF mice could address this question; and (d) we have not linked the mechanism of disease severity associated with defects in type I IFN signaling identified in mice with that of human patients having auto-Abs to IFNs and WNV encephalitis.

Nonetheless, our results correlating more severe WNV disease and the presence of neutralizing auto-Abs to type I IFNs extend our understanding of how acquired deficiencies of innate immune response pathways can predispose to severe viral infections and poor outcomes. Auto-Abs to type I IFNs are associated with a greater relative risk of severe COVID-19 and COVID-19-induced death^31,36^, severe COVID-19 vaccine breakthrough infections^34^, influenza pneumonia^35^, and detrimental outcomes after administration of a live-attenuated yellow fever virus vaccine^33^. Such studies suggest possible screening strategies and interventions in identified subjects who might be candidates for IFN-β therapy if administered as an early intervention, as was done successfully with a COVID-19 patient^37^. Possibly, administration of metabolites (*e.g*., short chain fatty acids) that promote barrier function or detoxify inflammatory components from the microbiota could be a complementary approach in selected populations to limit the adverse effects of loss of IFN signaling on the integrity of different barriers including the GI tract and the brain^74^.

## Methods

### Viruses

WNV New York 1999 (clone 382-99 [GenBank #AF196835]) was produced by electroporation of in vitro transcribed RNA into BHK21-15 cells^75^ and titered on Vero cells^76^. LCMV (C13 strain, gift of T. Egawa, Washington University in St. Louis) was propagated in BHK21 cells and titered on Vero cells.

### Human serum samples

Serum samples from asymptomatic WNV subjects identified through blood bank donations and a cohort of subjects with severe WNV neuroinvasive disease (Houston West Nile Cohort, Table 1)^44–46^ were obtained with written informed consent under approved protocols following the guidelines of the Human Investigations Committees of The University of Texas Health Science Center, Baylor College of Medicine, and Yale University School of Medicine, aliquoted, and then stored at -80^o^C^61,77^. The studies received Institutional Review Board approval from Baylor College of Medicine (H-30533). Severity of WNV infection was determined at the time of acute illness according to CDC guidelines (http://www.cdc.gov/ncidod/dvbid/westnile/clinicians/clindesc.htm) as previously described^14,78^. Asymptomatic, acutely infected subjects were identified via nucleic acid amplification testing by Gulf Coast Regional Blood Center^79^, and an absence of illness history was confirmed by study coordinators.

### Mice

Mouse experiments were carried out in accordance with the recommendations in the Guide for the Care and Use of Laboratory Animals of the National Institutes of Health. The protocols were approved by the Institutional Animal Care and Use Committee at the Washington University School of Medicine (Assurance #A3381-01). Wild-type C57BL/6 J (Jackson Laboratories, #000664) were obtained commercially. *Ifnar1*^-/-^ [B6(Cg)-*Ifnar1*^*tm1.2Ees*^/J, RRID: IMSR_JAX:028288^80^], *Ifngr*^-/-^ [B6.129 S7-*Ifngr1*^*tm1Agt*^/J, RRID: IMSR_JAX:003288^81^], *Ifnlr1*^-/-^ [*Ifnlr1*^*tm1Palu*^^82^], *Stat1*^-/-^ [B6.129 S(Cg)-*Stat1*^*tm1Dlv*^/J, RRID: IMSR_JAX:012606^83^], *Villin-Cre* [B6.Cg-Tg(Vil1-cre)997Gum/J, RRID: IMSR_JAX:004586^84^] and *Stat1*^*f/f*^ [B6;129S-*Stat1*^*tm1Mam*^/Mmjax, RRID: MMRRC_032054-JAX^85^] mice (all congenic on a C57BL/6 J background) were bred under pathogen-free conditions at Washington University. C57BL/6 J gnotobiotic mice were bred and housed at the Washington University Gnotobiotic Core Facility, and the GF status was confirmed through 16 S qPCR analysis of fecal samples (Charles River).

### Enteroid cultures

HIE cultures (J2, J2 *STAT1-KO* and J2 *IFNAR1-KO*) were previously described^86^ and purchased (Digestive Disease Core, Baylor College of Medicine). Mouse enteroids were established from duodenum and colon tissues obtained from wild-type and *Stat1*^*-/-*^ C57BL/6 J mice (Precision Animal Models and Organoids Core, Washington University). Enteroid cultures were maintained and passaged in Matrigel matrix (Corning, #354230) as multilobular three-dimensional (3D) cultures in 24-well plates supplemented with enteroid growth medium (WRNE, with Wnt, R-Spondin and Noggin growth factors in 50% L-WRN cell conditioned medium [CM for human organoids, and 50% L-WRN CM only for mouse enteroids). To generate confluent monolayers, the 3D enteroids were dissociated with TrypLE^TM^ Express (Thermo, #12604-013), filtered with a 40-μm cell strainer (Corning, #431750 and seeded onto collagen IV-coated 96-well plates as monolayers. For air-liquid interface (ALI) cultures, filtered organoid single cells were seeded as monolayers onto collagen IV-coated inserts of transwells (Corning, #3413), then cultured in WRNE medium supplied in both upper and lower chambers in transwells for two days, and the medium in the upper chambers were removed as the air phase for another two days.

### Virus infection of enteroid cultures

Following culture in growth medium for 24 h, monolayers were inoculated with viruses. Monolayers (approximately 2 ×10^4^ cells/well) were inoculated with WNV at different multiplicities of infection (MOI) (0.1 for human enteroids and 0.5 for mouse organoids, unless otherwise indicated), for 2 h at 37 °C in 5% CO_2_. The supernatants were harvested at 2 h after inoculation (input virus), and cells were then washed twice with complete medium without growth factors [CMGF(-)] to remove unbound viruses. Inoculated wells were then cultured in growth medium for specified time points to determine WNV replication kinetics. Each experiment was performed at least three times with three technical replicates in each condition.

### Focus-forming assay

Virus stocks were titered on Vero cells by focus-forming assay^76^. Vero cells were plated in 96-well plates and infected with serial dilutions of virus. At 16 h post-infection, cells were fixed, incubated with rat anti-WNV antibody, and stained with donkey anti-rat secondary antibody conjugated with horseradish peroxidase (HRP). Foci of infection were detected by addition of TrueBlue substrate (KPL, #5510-0030) and counted with a CTL Immunospot instrument, and infectious viral titers were calculated as focus-forming units (FFU) per mL.

### RNA extraction and RT-qPCR

Total RNA was extracted from cultured supernatants, organoid monolayers, tissue homogenates or sera samples using a MagMAX-96 Viral RNA Isolation Kit (Thermo, #AM1836) with the KingFisher™ Flex Purification System (Thermo). RT-qPCR reactions were performed using the TaqMan™ RNA-to-CT™ *1-Step* Kit (Applied Biosystems, #4392938) and the QuantStudio 6 Flex Real-Time PCR System. The WNV primer set includes: Forward primer (5’- TCA GCG ATC TCT CCA CCA AAG -3’), Reverse primer (5’- GGG TCA GCA CGT TTG TCA TTG -3’) and probe (/56-FAM/TGC CCG ACC ATG GGA GAA GCT C/36-TAMSp/). The LCMV primer set includes: Forward primer (5’- TGC CTG ACC AAA TGG ATG ATT -3’), reverse primer (5’- CTG CTG TGT TCC CGA AAC ACT -3’) and Taqman probe (/56-FAM/TTG CTG CAG AGC TT/36-TAMSp/). A standard curve based on quantified WNV and LCMV samples were used to quantitate viral genome equivalents in experimental samples.

### Mouse infection and tissue collection

For WNV infection studies in wild-type C57BL/6 mice, 9-10-week-old male mice were inoculated with 10^2^ FFU WNV in 50 μL via footpad injection, following anesthesia with xylazine and ketamine hydrocloride. In some experiments, mice were administered mAbs one day prior to infection: anti-IFNAR1 antibody (Leinco #I-401, clone MAR1-5A3) or mouse IgG1 isotype control antibody (Leinco #I-117, clone HKSP84) via intraperitoneal (2 mg/mouse) or retroorbital (1.6 mg/mouse) injection, with or without the addition of either anti-TNF-α antibody (200 μg, Biolegend #506352, clone MP6-XT22) or rat IgG1 isotype control antibody (200 μg, Invitrogen, #14-4301-85). For experiments with *Stat1*^-/-^ and *Ifnar1*^*-/-*^ mice, both sexes of mice were tested. Anti-TNF-α antibody (200 μg, Biolegend #506352, clone MP6-XT22) was administered by intraperitoneal injection one day prior to WNV infection^66^ to delay early lethality. SPF mice were weighed daily, whereas GF mice were weighed at 0 and 5 dpi. At the indicated times post-infection, mice were administered terminal anesthesia, followed by blood collection via cardiac puncture and extensive perfusion with PBS. Tissues including the GI tract, liver, spleen, spinal cord, and brain were collected, weighed, homogenized in 500 µL of ice-cold PBS (6000 RPM for 60 sec; Roche MagnaLyser) and then clarified by centrifugation (14,000 x g, 4^o^C for 10 min) prior to downstream assays.

### Histological staining and analysis

Liver specimens were fixed in 4% PFA at 4^o^C, dehydrated in 70% ethanol, cleared with xylenes, and embedded in paraffin. Microtome-sectioned samples were stained with hematoxylin and eosin following standard protocols. Analysis of liver tissue was performed in a blinded manner using ImageJ software. The percentage of liver necrosis was determined by quantitating the number of pixels within necrotic lesions divided by the total number of pixels in the liver parenchyma. The severity score of ballooned hepatocytes was determined as follows: score 0 = absent; score 1 = < 5%; score 2 = 5–10%; score 3 = 10–20%; score 4 = 20–50%; score 5 = > 50%.

### Multiplex analysis of cytokines

This study used Luminex xMAP technology for multiplexed quantification of mouse cytokines, chemokines, and growth factors. The multiplexing analysis was performed using the Luminex™ 200 system by Eve Technologies Corp. Thirty-two markers were concurrently measured in the samples using Eve Technologies’ Mouse Cytokine 32-Plex Discovery Assay® (MilliporeSigma, Burlington, Massachusetts, USA) according to the manufacturer’s protocol. The 32-plex consisted of Eotaxin, G-CSF, GM-CSF, IFNγ, IL-1α, IL-1β, IL-2, IL-3, IL-4, IL-5, IL-6, IL-7, IL-9, IL-10, IL-12(p40), IL-12(p70), IL-13, IL-15, IL-17, IP-10, KC, LIF, LIX, MCP-1, M-CSF, MIG, MIP-1α, MIP-1β, MIP-2, RANTES, TNFα, and VEGF. Assay sensitivities of these markers range from 0.3 to 30.6 pg/mL for the 32-plex. Individual analyte sensitivity values are available in the MilliporeSigma MILLIPLEX® MAP protocol.

### Immunofluorescence staining of GI tracts and organoid monolayers

Sections of GI tract tissue were opened along the mesenteric border, pinned to Sylgard silicon plates, and fixed in 4% paraformaldehyde (PFA, Electron Microscopy Sciences, #15713-S) overnight at 4^o^C. For staining of WNV antigen, PFA-fixed GI segments were cryopreseserved in 30% glucose overnight, embedded in Tissue-Tek O.C.T. Compound (Sakura Finetek USA, #4583), frozen, and then sectioned to 10-μm depth by Cryostat (Leica, #CM1850). Slides were blocked for 1 h at room temperature in Tris-buffered saline (TBS) solution containing 1% bovine serum albumin (BSA), 5% normal donkey serum (Sigma, #D9663), and 0.1% Triton X-100 (Sigma, #T8787). Sections were incubated overnight at 4 ^o^C with rat anti-WNV hyperimmune serum (1:750^87^,) and rabbit anti-EpCAM polyclonal serum (1:2000, Abcam #ab71916) or rabbit anti-STAT1 monoclonal antibody (1:1000, Cell Signaling #14994) diluted in 1X TBS containing 1% BSA, 3% normal donkey serum, and 0.1% Triton X-100. After three rinses with PBS, the sections were incubated with secondary antibodies in the dark for 2 h at room temperature including AF594-conjugated donkey anti-rat antibody (1:1000, ThermoFisher #A-21209), AF647-conjugated donkey anti-rabbit antibody (1:1000, ThermoFisher #A-31573), and fluorescein-conjugated Ulex Europaeus Agglutinin I (UEA-1, 1:2000, ThermoFisher #L32476) diluted in TBS containing 1% BSA, 3% normal donkey serum, and 0.1% Triton X-100. Following three rinses with PBS and counter-staining with Hoechst 33258 dye (1:20,000 in 1X PBS, ThermoFisher #H3569) and two additional rinses, tissue sections were mounted in ProLong Glass Antifade Mountant (Invitrogen #P36980) and stored in the dark at 4^o^C until imaging. For staining of organoid ALI monolayers in transwells, the membrane was rinsed with PBS and fixed by 100% methanol at -20^o^C for 20 min, then blocked with blocking buffer (1X PBS with 2% normal donkey serum, 1% BSA, 0.1% cold fish gelatin, 0.1% Triton X-100, 0.05% Tween 20 and 0.05% NaN_3_). The membrane was carefully cut out with a blade and transferred into a new well in a 24-well dish, followed by staining with primary antibodies including mouse anti-ZO-1 monoclonal antibody (1:1000, ThermoFisher #33-9100) in 1X PBS with 1% BSA, 0.1% cold fish gelatin and 0.05% NaN_3_, and secondary antibody including AF488-conjugated donkey anti-mouse antibody (1:1000, ThermoFisher #A-21202) in 1X PBS. After staining the membrane was mounted in ProLong Glass Antifade Mountant and stored in the dark at 4^o^C until imaging as well. All images were acquired using a Zeiss LSM 880 Confocal Laser Scanning Microscope 20x (NA 0.8) objective. The WNV antigen signals in all images was quantified by Fiji ImageJ software. Each data point was derived from measurements of 3 to 5 independent fields per mouse per GI tract segment.

### GI tract permeability assay

Intestinal permeability was measured by detecting translocation of dextran from the lumen of the GI tract into the blood. Briefly, at 3 or 5 dpi, mice were gavaged orally with 100 µL of 60 mg/mL of dextrans of different molecular weights conjugated to different fluorescent dyes: Cascade Blue, (10 kDa, Thermo, #D1976), Tetramethylrhodamine (70 kDa, Thermo, #D1818) and FITC (250 kDa, Sigma-Aldrich, #FD250S). At 3 h after dextran administration, mice were administered terminal anesthesia with ketamine/xylazine, and blood was collected by cardiac puncture into serum separator tubes (BD Microtainer, #365967) and centrifuged (1500x *g*, 10 min at 4^o^C). Fluorescence (360, 490 and 550 nm wavelength absorbance endpoints) was measured with a BioTek Synergy H1 plate reader, and dextran concentrations in the sera were calculated based on standard curves. To evaluate for accumulation of translocated dextrans into the brain using confocal microscopy imaging, brain tissues were fixed in 4% PFA overnight at 4^o^C, embedded in Tissue-Tek O.C.T. Compound (Sakura Finetek USA, #4583), sectioned to 10-μm, and counterstained with Hoechst 33258 dye. Some samples were also stained for WNV antigen as described above. Images were acquired using a Zeiss LSM 880 Confocal Laser Scanning Microscope 20x (NA 0.8) objective. Each data point was derived from measurements of 3 to 5 independent fields per mouse per brain.

### Bacteria culture

At the time of necropsy, approximately 500 μL of blood was sterilely collected from each mouse via cardiac puncture and transferred to sterile collection tubes containing EDTA (Becton Dickinson, #365974). Blood was diluted in sterile 1x DMEM (no antibiotics or FBS) and serial dilutions were made (undiluted, 1:10 and 1:100). Approximately 50 µL of blood and diluted samples from each mouse was plated on pre-warmed sheep blood agar plates (Fisher Scientific, # B21261) using L-shaped spreaders. Plates were incubated at 37^o^C overnight, and the number of colonies was enumerated on the following day.

### BBB permeability assay

BBB integrity was evaluated by examining extravasation of dextrans of different sizes from the blood into the brain. Briefly, mice were anesthesized and administered by intravenous injection 100 µL of 40 mg/mL dextrans conjugated to FITC (250 kDa, Sigma-Aldrich, #FD250S) or tetramethylrhodamine (TRITC) (70 kDa, Thermo, #D1818). After 25 min, mice were administered terminal anesthesia with ketamine/xylazine. Subsequently, brains were harvested, fixed in 4% PFA overnight at 4^o^C, cryoprotected with 30% sucrose over 48 h, embedded in Tissue-Tek O.C.T. Compound (Sakura Finetek USA, #4583), frozen, and then sectioned to 30-μm depth by Cryostat (Leica, #CM1850). Sections were counterstained with Hoechst 33258 (1:10,000 in 1X PBS, ThermoFisher #H3569), mounted in ProLong Glass Antifade Mountant, and images were acquired using Zeiss Laser Scanning disk microscope 10x objective (NA 0.3), stitched (ZEN Blue software, Zeiss), and processed and analyzed by Fiji software (https://fiji.sc/Fiji). Quantification was performed by calculating the area of dextran translocation as a fraction of the total area of delineated brain region.

### Brain microglia and macrophage staining

Mouse brains were harvested, fixed in 4% PFA overnight at 4^o^C, cryoprotected with 30% sucrose over 48 h, embedded in Tissue-Tek O.C.T. Compound (Sakura Finetek USA, #4583), frozen, and then sectioned to 10-μm depth by Cryostat (Leica, #CM1850). Slides were blocked for 1 h at room temperature in Tris-buffered saline (TBS) solution containing 1% bovine serum albumin (BSA), 5% normal donkey serum (Sigma, #D9663), and 0.1% Triton X-100 (Sigma, #T8787). Sections were incubated overnight at 4^o^C with rabbit anti-Iba1 antibody (1:500, Abcam #ab178846) diluted in 1X TBS containing 1% BSA, 3% normal donkey serum, and 0.1% Triton X-100. After three rinses with PBS, the sections were incubated in the dark for 2 h at room temperature with AF647-conjugated donkey anti-rabbit antibody (1:1,000, ThermoFisher #A-31573) diluted in TBS containing 1% BSA, 3% normal donkey serum, and 0.1% Triton X-100. Following three rinses with PBS and counter-staining with Hoechst 33258 dye (1:20,000 in 1X PBS, ThermoFisher #H3569) and two additional rinses, tissue sections were mounted in ProLong Glass Antifade Mountant (Invitrogen #P36980) and stored in the dark at 4 ^o^C until imaging. All images were acquired using a Zeiss LSM 880 Confocal Laser Scanning Microscope 20x (NA 0.8) objective Image processing and analysis was performed using ImageJ. The number of Iba1^+^ cells were counted per field. The area of Iba1 staining was quantified by converting images to binary using a threshold tool from at least 20 microglia/macrophage cells in each field and then dividing by the number of microglia/macrophage nuclei counted. Each data point was derived from measurements of at least 3 independent fields per mouse brain.

### Anti-type I IFN ELISA

Assays were performed in 96-well ELISA plates (MaxiSorp; Thermo Fisher Scientific). The plates were coated with 2 μg/mL of recombinant human IFN-α2a (Milteny Biotec, #130-093-874), IFN-β1 (Milteny Biotec, #130-107-888) or IFN-ω (Sigma, #SRP3061) in ELISA coating buffer (Na_2_CO_3_ and NaHCO_3_, 0.05 M Carbonate-Bicarbonate, pH 9.6) overnight at 4^o^C. Plates were rinsed eight times with PBST (PBS with 0.05% Tween-20), blocked after incubation with 5% human serum albumin (Sigma, #A1653) in PBST, rinsed eight times, and then incubated with serum from WNV patients (1:25), positive serum controls (1:100, obtained from S. Holland, National Institutes of Health) and negative naïve serum controls (1:25) for 2 h at room temperature. Plates were rinsed 8 times with PBST and then incubated with horseradish peroxidase (HRP)-conjugated Fc-specific goat anti-human IgG/IgA/IgM (1:10,000, Nordic Immunological Laboratories #GAHu/Ig(Fc)/PO) for 2 h at room temperature. After rinsing eight times with PBST, TMB (3,3’,5,5’-Tetramethylbenzidine, Thermo #34029) substrate was added as 100 μL per well for 5 min and stopped with 2 N H_2_SO_4_ (100 μL/well), and the optical density (OD) at 450 nm wavelength was measured. An OD value above the average of all negative samples plus six times their standard deviation (Background Avg + 6 x SD) was defined as a positive signal.

### IFN neutralization assay

The neutralizing activity against IFN-α or IFN-ω was determined using a published reporter luciferase activity^32^. Briefly, HEK293T cells were transfected with a *Firefly* luciferase reporter plasmid under the control of the human ISRE promoter in the pGL4.45 backbone using X-tremeGene9 transfection reagent (Sigma-Aldrich, #6365779001), with the plasmid constitutively expressing *Renilla* luciferase for normalization (pRL-SV40). One day later, transfected cells cultured in DMEM (Thermo Fisher Scientific, #11965-084) supplemented with 2% FBS and 10% asymptomatic control or neuroinvasive patient serum/plasma (after heat-inactivation at 56 °C, for 20 min) were either left unstimulated or were stimulated with IFN-α2 (Miltenyi Biotec, #130-108-984) or IFN-ω (Peprotech, #300-02 J) at 10 ng/mL for 16 h at 37 °C. Cells were lysed, and luciferase levels were measured with the Dual-Luciferase® Reporter 1000 assay kit (Promega, #E1980), according to the manufacturer’s protocol. Luminescence intensity was measured with a VICTOR Nivo Multimode Microplate Reader (PerkinElmer Life Sciences, USA). *Firefly* luciferase activity values were normalized against *Renilla* luciferase activity values. ISRE induction values were then normalized against the median induction values for non-neutralizing samples and expressed as percentages. Samples were considered neutralizing if the fold induction was below 15% of the median values for non-neutralizing controls.

### IFN neutralization in human enteroid cultures

Wild-type or *IFNAR1-KO* human enteroids were pretreated with either 20 ng/mL of IFN-α2 for 8 h directly or IFN-α2 in the presence of 10% (v/v) patient serum sample for 8 h. The treated wells were then inoculated 2 ×10^3^ FFU of LCMV clone 13 (MOI of 0.1) for 2 h at 37 °C in 5% CO_2_. The supernatants with virus inoculum then were harvested as input, and cells were washed twice with medium to remove unbound viruses. Inoculated wells were then supplemented with 100 μL/well of WRNE medium and cultured for 48 h at 37 °C in 5% CO_2_. Samples were assessed for LCMV replication by RT-qPCR. In WNV infection studies, wild-type human enteroids were pretreated with 100 pg/mL of IFN-α2 for 8 h with or without 10% (v/v) patient sera for 8 h. The treated wells were then inoculated 2 ×10^3^ FFU of WNV (MOI of 0.1) for 2 h at 37 °C in 5% CO_2_. The supernatants were harvested as a measure of input virus, and cells were washed twice with medium to remove unbound viruses. Wells were then supplemented with 100 μL/well of WRNE medium and cultured for 72 h at 37 °C in 5% CO_2_. Samples were collected and assessed for WNV replication by RT-qPCR.

### Statistical analysis

Experimental animals were randomized throughout the study, and scoring of immunofluorescence and histology images was performed in a blinded manner. Statistical analyses were performed with Prism 9.0. Mann-Whitney, one-way ANOVA, and two-way ANOVA tests were used to determine significance with Bonferroni correction for multiple comparisons. Details of statistical tests, number of animals, mean values, and comparison groups are included in the Figure Legends. In the initial trials, two GF mice that received anti-IFNAR1 antibodies via intraperitoneal injection were excluded from analysis after testing of sera and tissues failed to reveal detectable WNV RNA in tissues at 5 dpi, consistent with unsuccessful systemic delivery of anti-IFNAR1. All subsequent GF mice received anti-IFNAR1 antibodies via retroorbital injection and demonstrated markedly elevated levels of WNV RNA, as anticipated.

### Reporting summary

Further information on research design is available in the Nature Portfolio Reporting Summary linked to this article.

### Supplementary information

Supplementary Information

Reporting Summary

### Source data

Source Files

## Data Availability

All data supporting the findings of this study are available within the main text and supplemental data. Source data for main and supplemental figures are provided with this paper. Source data are provided with this paper.
