# Supplementary material for: Blockade of interferon signaling decreases gut barrier integrity and promotes severe West Nile virus disease
Source: Nat Commun. 2023 Sep 25;14:5973. doi: 10.1038/s41467-023-41600-3 (PMC10520062; doi:10.1038/s41467-023-41600-3)
Supplement: Supplementary file 1 — Supplementary Information [file 41467_2023_41600_MOESM1_ESM.pdf]

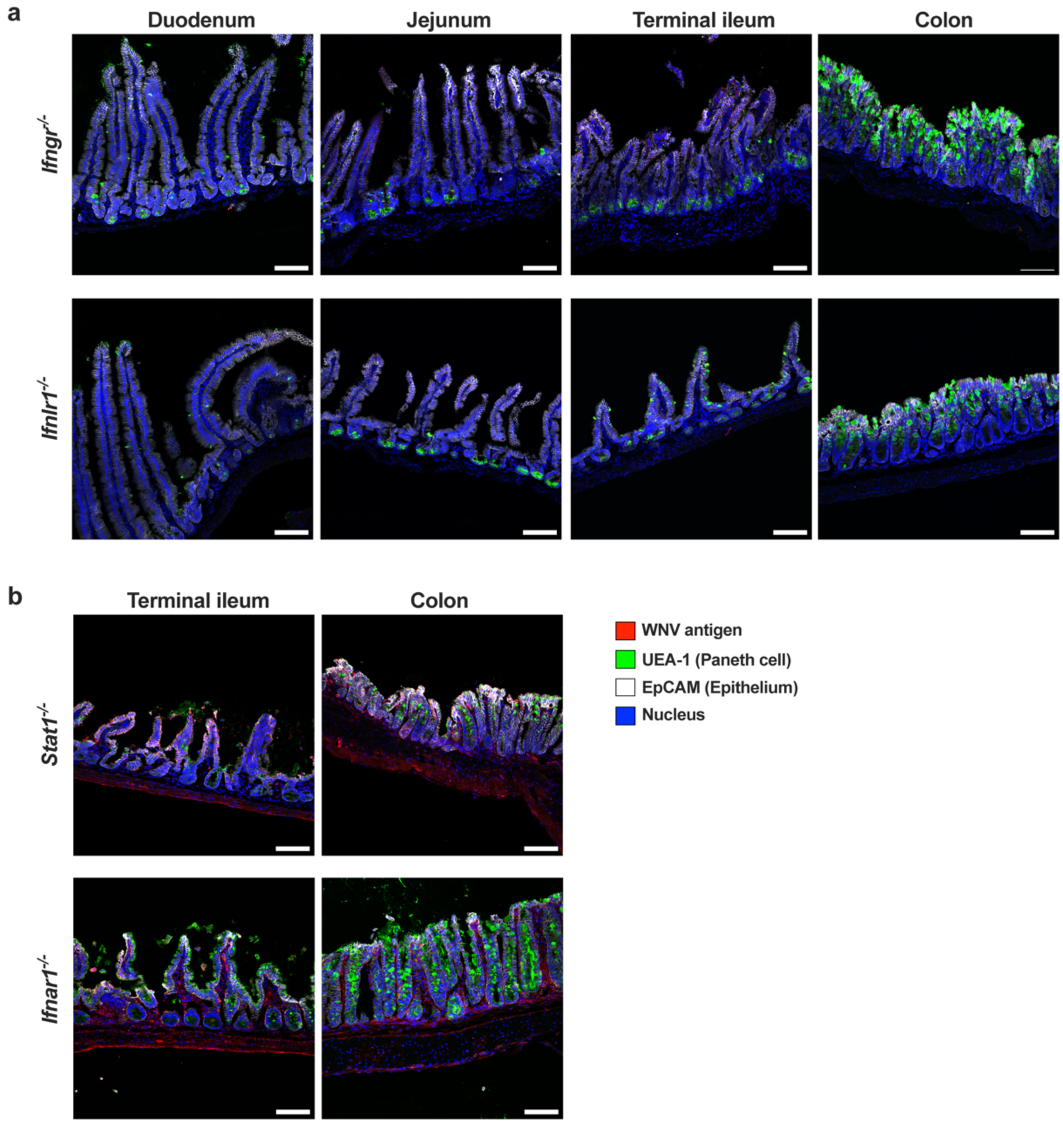

Supplementary Figure 1. WNV infection in the GI tract of SPF mice with *Ifngr1*-, *Ifnlr1*-, *Ifnar1*- or *Stat1*-deficiencies. The indicated congenic strains of C57BL/6J SPF mice were inoculated subcutaneously in the footpad with 10^2 FFU of WNV, and sections of the GI tract were harvested at 5 dpi. Immunofluorescent confocal images are shown of (**a**) sections along the GI tract for WNV-infected *Ifngr*$^{-/-}$ or *Ifnlr1*$^{-/-}$ mice (2 experiments with n = 8 mice per genotype), and (**b**) ileum and colon sections from *Stat1*$^{-/-}$ or *Ifnar1*$^{-/-}$ mice (3 experiments with n = 8 mice per genotype). Images show WNV antigen (red), Paneth and goblet cells (UEA-1, green), EpCAM (white), and nuclei (Hoechst 33258, blue). Scale bar, 100 μm.

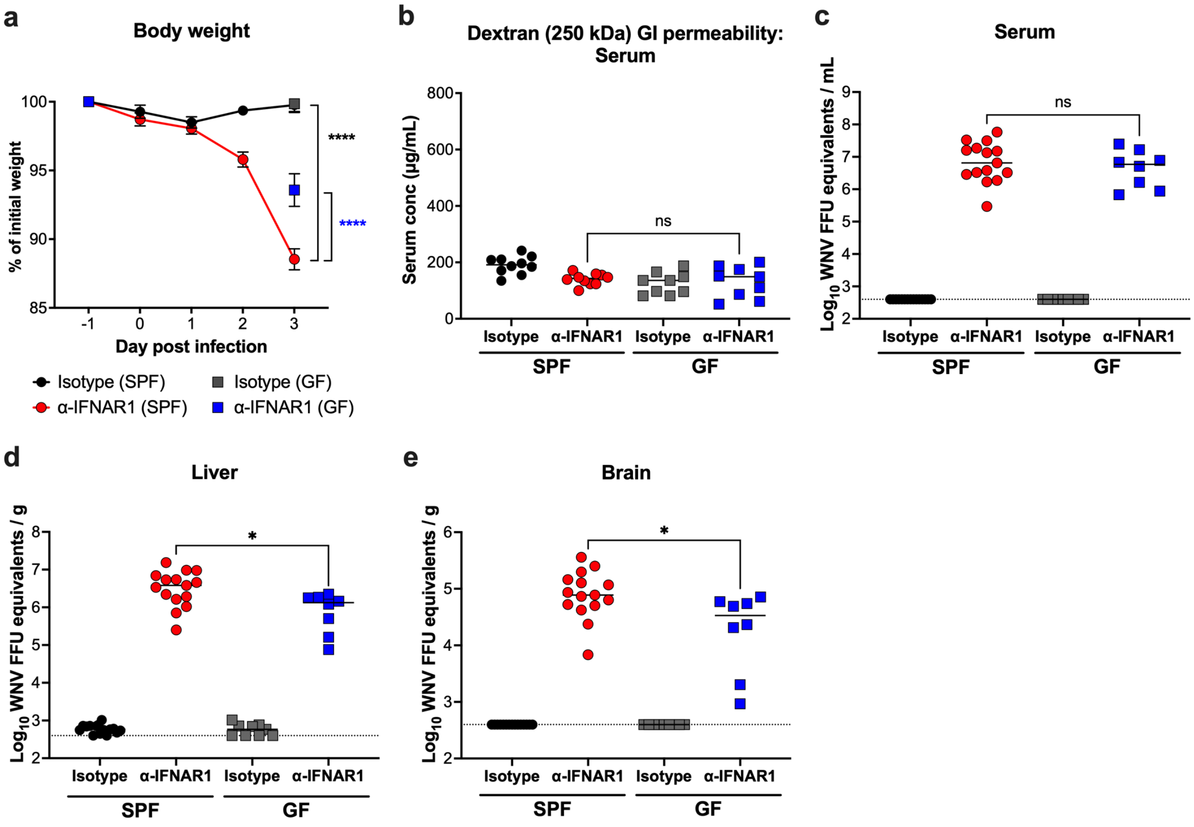

Supplementary Figure 2. The effect of type I IFN signaling blockade on GI permeability and WNV viral burden at 3 dpi. SPF or GF mice treated with either isotype control or blocking IFNAR1 antibodies were infected with WNV. At 3 dpi, animals were administered 250 kDa FITC-dextran by oral gavage. **a**, Body weight measurements. Bars indicate mean ± standard error of the mean (2 experiments, n = 15 for SPF mice per group and n = 9 for GF mice per group). **b**, GI tract permeability of 250 kDa FITC-dextran was measured (2 experiments, from left to right, n = 10, 10, 9, and 9 mice per group). **c-e**, WNV RNA levels in serum (**c**), liver (**d**), and brain (**e**) were determined by RT-qPCR. Bars indicate geometric means; dotted lines show LOD (3 experiments, from left to right, n = 15, 15, 9, and 8 mice per group. Statistical analysis: two-way ANOVA with Šídák's post-test: ****P < 0.0001 (**a**); two-tailed Mann-Whitney test: ns, not significant (**b-c**), *P = 0.0105 (**d**); *P = 0.0105 (**e**).

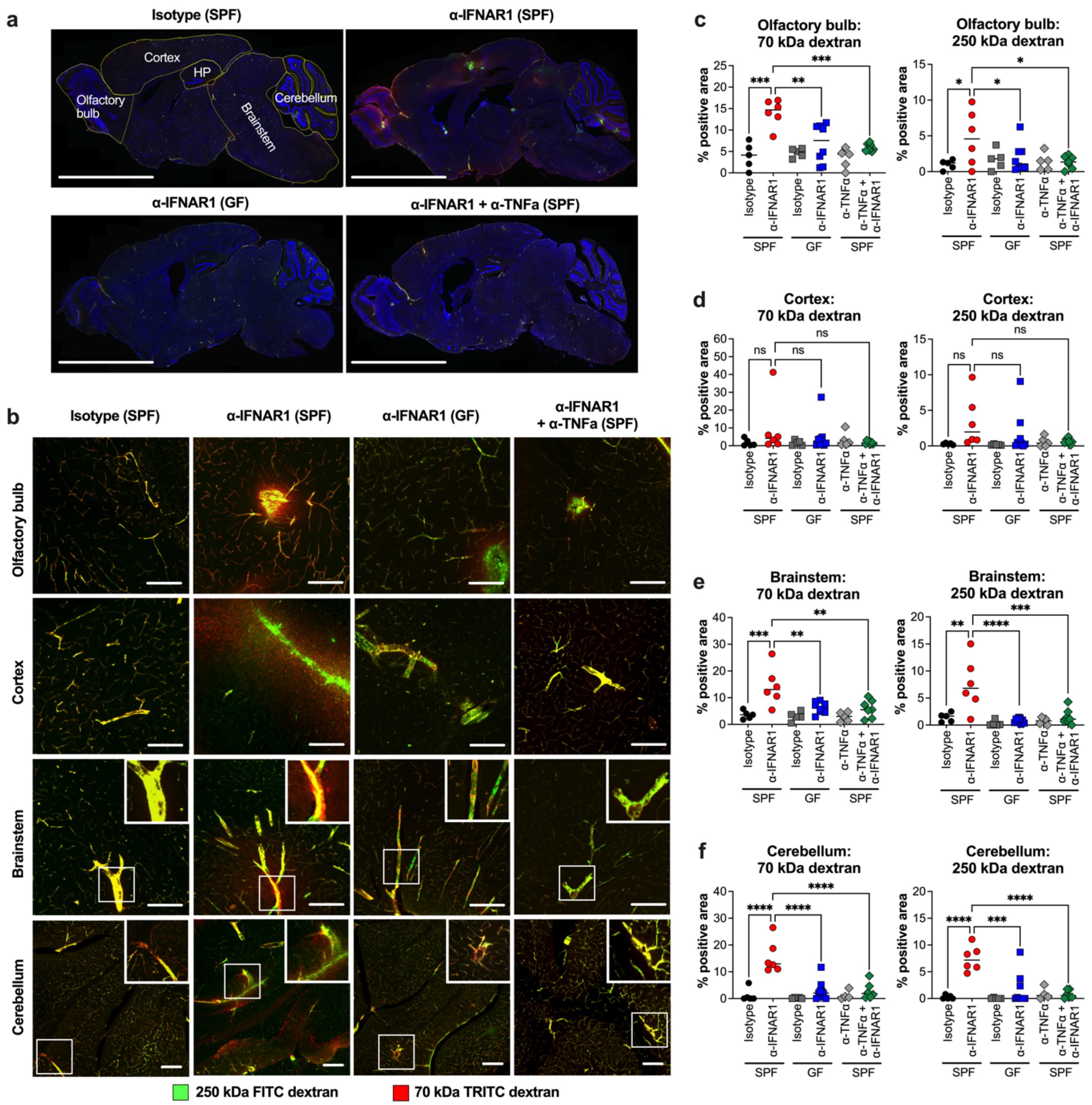

Supplementary Figure 3. Blockade of type I IFN signaling compromises the BBB of WNV infected mice and is ameliorated in germ-free mice. Wild-type SPF C57BL/6J mice treated with either isotype control or blocking anti-IFNAR1 antibodies were inoculated subcutaneously in the footpad with 10^2 FFU of WNV, and brains were harvested at 5 dpi after intravenous injection of 70 kDa TRITC and 250 kDa FITC dextrans. **a,** Schematic of brain regions used for quantification of FITC or TRITC positive area from SPF isotype, SPF anti(α)-IFNAR1, GF anti(α)-IFNAR1, and SPF anti(α)-IFNAR1/anti(α)-TNFα treated mice. Scale bar, 5 mm. **b,** Imaging of sections shows 250 kDa FITC (green) and 70 kDa TRITC (red) within the vasculature and translocated into the indicated brain parenchyma regions. Boxed insets focusing on blood vessels are shown at higher magnification for some sections. Scale bars,100 μm. **c-f,** Accumulation of translocated dextrans was quantified as

the percentage of FITC or TRITC positive areas in the olfactory bulb (**c**), cortex (**d**), brainstem (**e**), or cerebellum (**f**). Bars indicate mean values. Data are from 2 experiments with (from left to right) n = 5, 6, 5, 8, 5, and 7 mice per group (**c**); n = 5, 6, 5, 7, 5, and 9 mice per group (**d**); n = 5, 6, 5, 7, 5, and 9 mice per group (**e**); n = 5, 6, 4, 6, 5, and 9 mice per group (**f**). Statistical analysis: one-way ANOVA with Dunnett's post-test, from left to right: ***$P = 0.0001$, **$P = 0.0013$, ***$P = 0.0005$, *$P = 0.0328$ (**c**); ns, not significant, (**d**); ***$P = 0.0005$, **$P = 0.0025$, **$P = 0.0025$, **$P = 0.0011$, ****$P < 0.0001$, ***$P = 0.0005$ (**e**); ****$P < 0.0001$, ***$P = 0.0001$ (**f**).

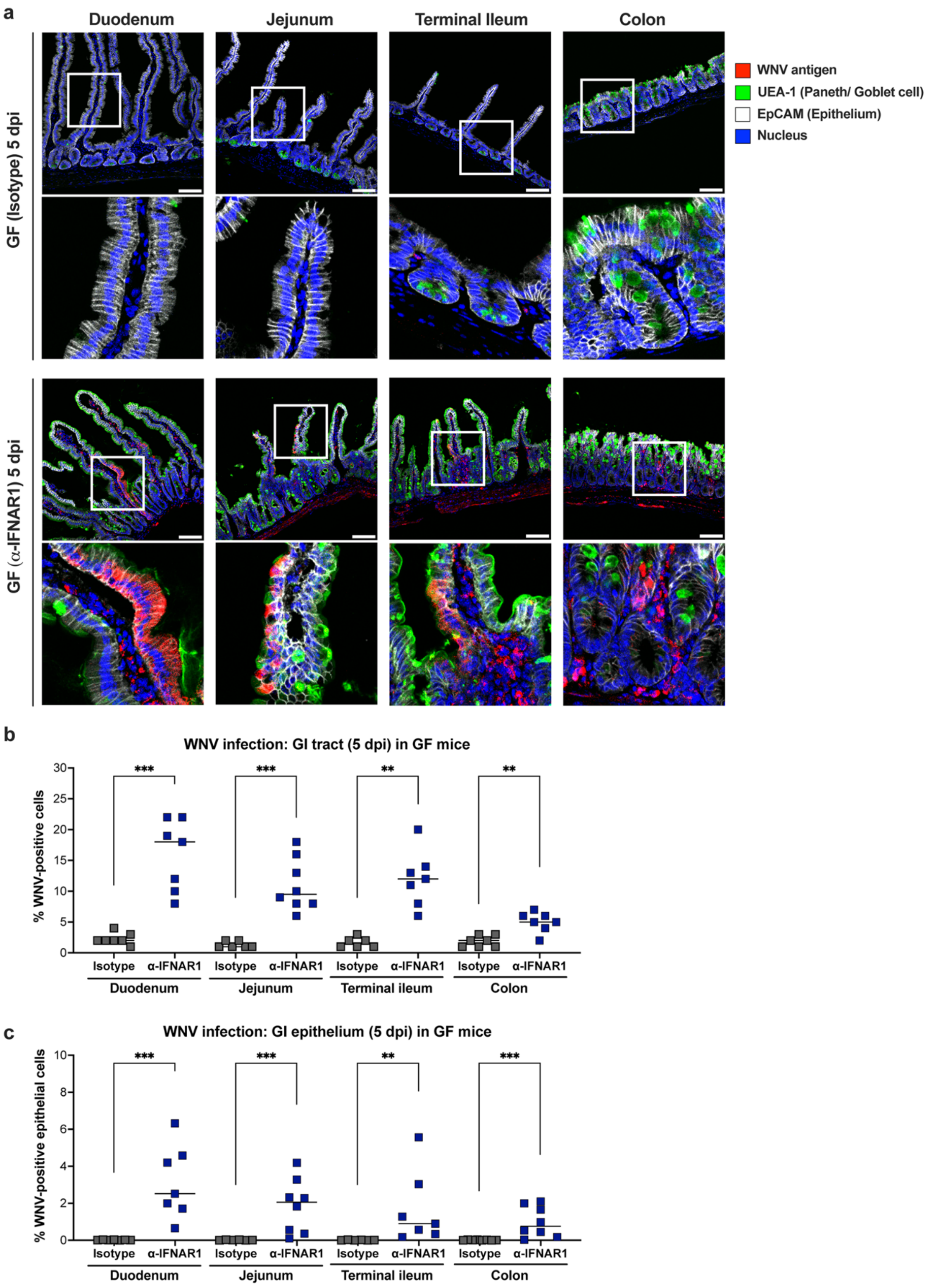

Supplementary Figure 4. WNV infection in the GI tract of GF mice with IFNAR1 blockade. GF C57BL/6J mice treated with either isotype control or anti-IFNAR1 antibodies were inoculated subcutaneously in the footpad with 10^2 FFU of WNV. **a,** Immunofluorescence confocal microscopy

imaging of sections of indicated regions of the GI tract at 5 dpi; WNV antigen (red), Paneth and goblet cells (UEA-1, green), EpCAM (white), and nuclei (Hoechst 33258, blue). Scale bar, 100 µm. Data are representative of 3 experiments with n = 11 mice per group. **b-c**, Quantitation of WNV antigen-positive cells in different regions along the GI tract was determined as a percentage of total Hoechst 33258-positive cells per field (**b**) or as a percentage of total EpCAM-positive epithelial cells per field (**c**). Bars indicate mean values. Data are from 3 experiments, from left to right, with n = 7, 7, 6, 8, 6, 7, 7, and 7 mice per group. Statistical analysis: two-tailed Mann-Whitney test, from left to right: ****P* = 0.0006, ****P* = 0.0003, ***P* = 0.0012, ***P* = 0.0035 (**b**); ****P* = 0.0006, ****P* = 0.0007, ***P* = 0.0012, ****P* = 0.0005 (**c**).

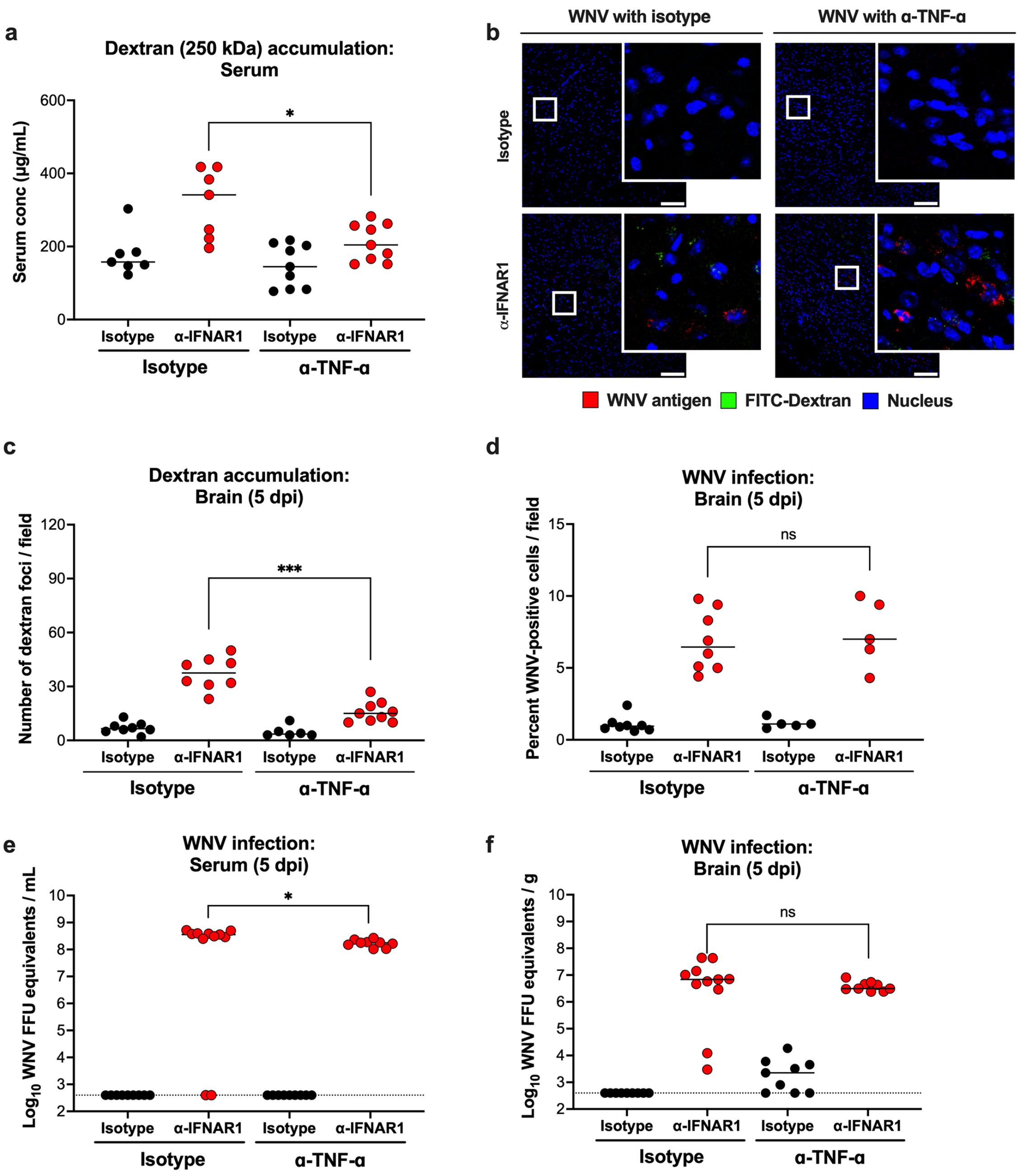

Supplementary Figure 5. TNF-α blockade rescues GI tract permeability in SPF mice treated with anti-IFNAR1 antibodies. SPF mice were treated with isotype control or blocking anti-IFNAR1 antibodies, along with blocking anti-TNF-α antibodies, and then infected with WNV. At 5 dpi, mice were gavaged orally with fluorescently-labelled 250 kDa dextran. **a**, Concentration of fluorescently-labelled dextran in sera 3 h after oral gavage. Bars indicate mean values. Data are from 2 experiments, from left to right, with n = 7, 7, 9, and 9 mice per group. **b-d**, Fluorescently-labelled dextran (FITC, green), WNV-antigen (red), and nuclei (Hoechst 33258, blue) in brain tissue sections were imaged by confocal microscopy (**b**) and quantitated (**c-d**). Scale bar, 100 µm. High-power insets are shown from the boxed regions. Bars indicate mean values. Data are from 2

experiments, from left to right, with n = 8, 8, 6, and 9 mice per group (**c**) and with n = 8, 8, 5 and 5 mice per group (**d**). **e-f**, WNV RNA levels in serum (**e**) and brain homogenates (**f**). Bars illustrate geometric means, dotted lines show LOD. Data are from 2 experiments, from left to right, with n = 9, 11, 9, and 9 mice per group. Statistical analysis: (**a, c-f**) two-tailed Mann–Whitney test: **P* = 0.0404 (**a**); ****P* = 0.0002 (**c**); ns, not significant (**d**); **P* = 0.0196 (**e**); ns, not significant (**f**).

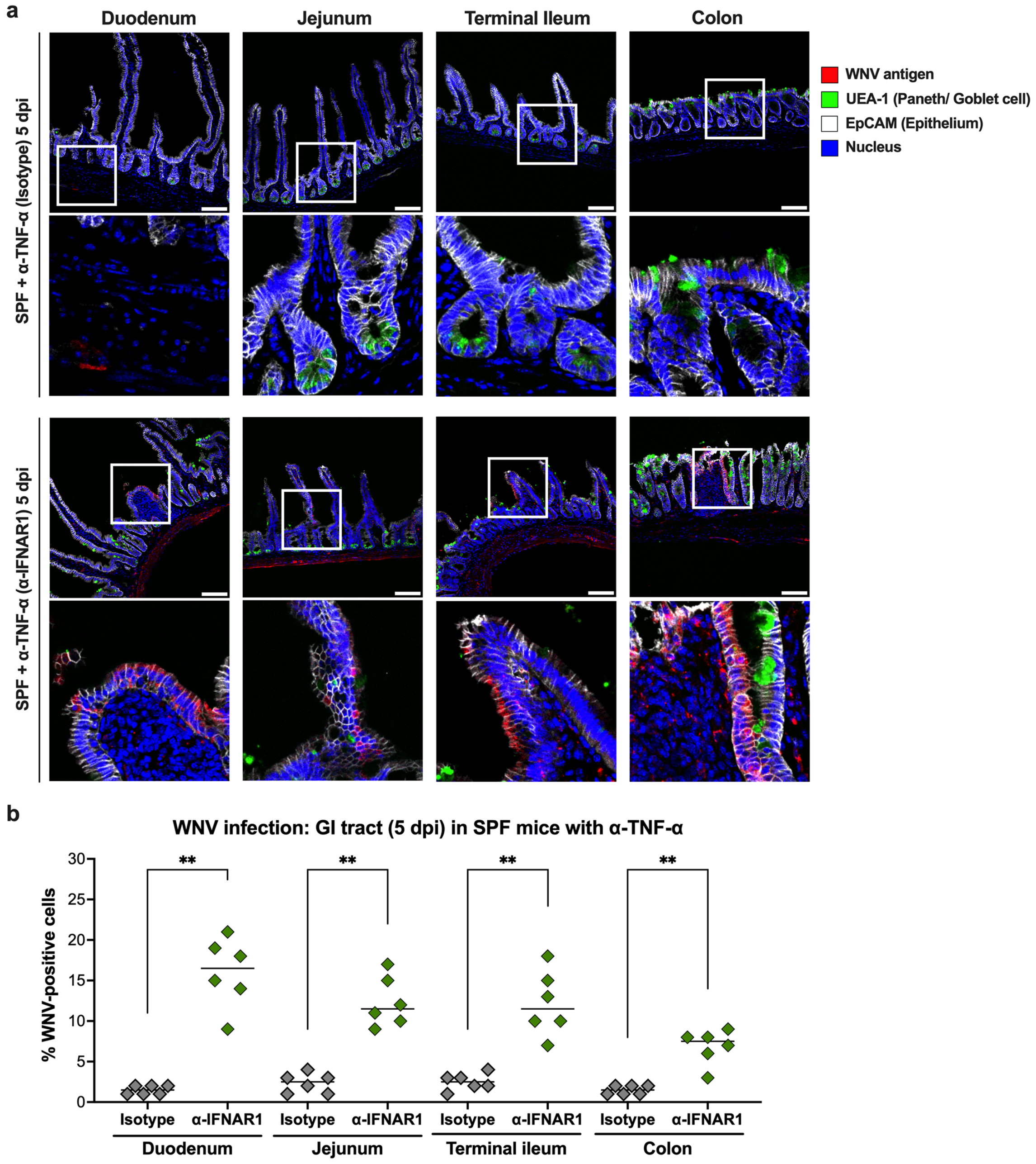

Supplementary Figure 6. TNF-α blockade does not alter WNV infection in the GI tract of SPF mice treated with anti-IFNAR1 antibodies. C57BL/6J SPF mice treated with anti-TNF-α and isotype control or anti-IFNAR1 antibodies were inoculated subcutaneously in the footpad with 10^2 FFU of WNV. **a**, Immunofluorescence confocal microscopy imaging of sections of indicated regions of the GI tract at 5 dpi; WNV antigen (red), Paneth and goblet cells (UEA-1, green), EpCAM (white), and nuclei (Hoechst 33258, blue). Scale bar, 100 µm. Data are representative of 2 experiments with n = 6 mice per group. **b**, Quantitation of WNV antigen-positive cells in different regions of the GI tract was determined as a percentage of total Hoechst 33258-positive cells per field. Bars indicate mean values. Data are from 2 experiments with n = 6 mice per group. Statistical analysis: two-tailed Mann-Whitney test: $^{**}P = 0.0022$.

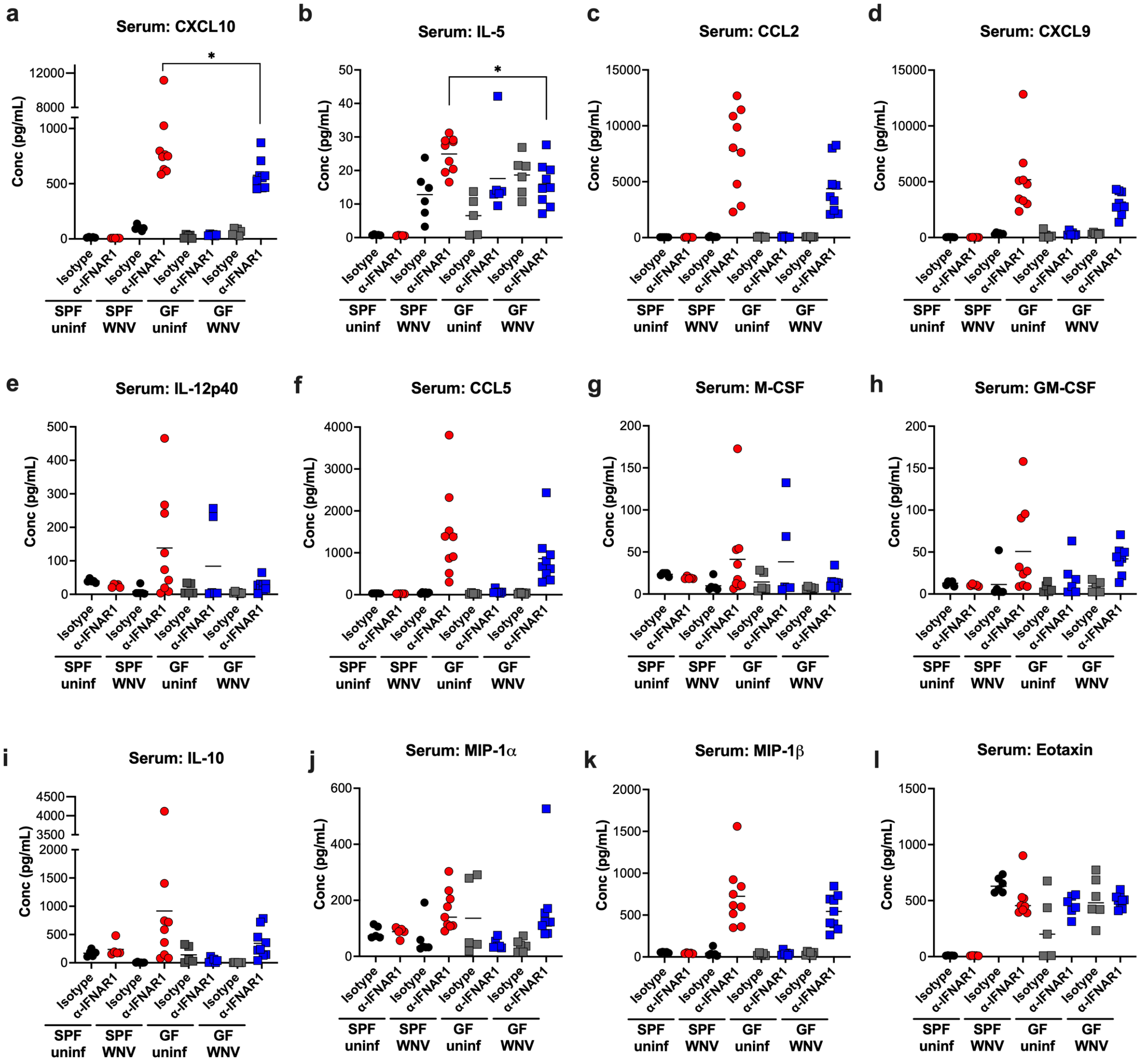

Supplementary Figure 7. Cytokine and chemokine levels in serum in uninfected and WNV-infected SPF and GF mice. a-l, GF and SPF mice were treated with isotype control or anti-IFNAR1 antibody. Some of the animals were then infected with WNV via subcutaneous inoculation. At 5 dpi, serum was harvested, and cytokines and chemokines were measured using a multiplexed assay (see Methods). Serum levels of CXCL10 (**a**), IL-5 (**b**), CCL2 (**c**), CXCL9 (**d**), IL-12p40 (**e**), CCL5 (**f**), M-CSF (**g**), GM-CSF (**h**), IL-10 (**i**), MIP-1α (**j**), MIP-1β (**k**), and eoxtaxin (**l**) are shown. Data are from 2 independent experiments with bars indicating the mean values; from left to right, n = 5, 5, 6, 9, 5, 6, 6, and 9 mice per treatment group. Statistical analyses are for comparisons between WNV-infected anti-IFNAR1-treated SPF and GF mice: two-tailed Mann-Whitney test with Bonferroni correction; *P = 0.034 (**a**), *P = 0.029 (**b**).

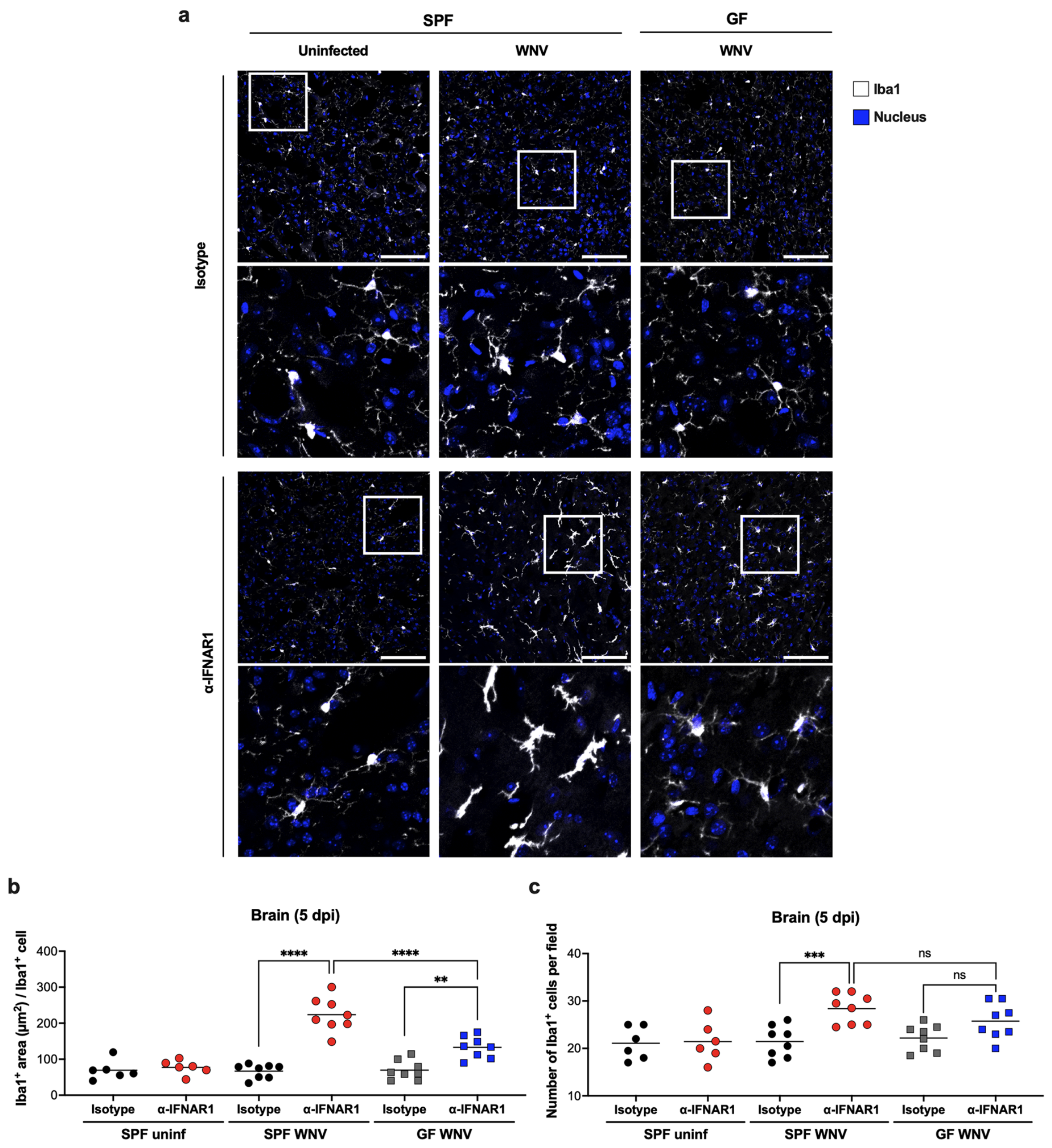

Supplementary Figure 8. Microglia/macrophage activation in brains of WNV-infected SPF and GF mice. SPF or GF mice were treated with either isotype control or anti-IFNAR1 antibodies and then inoculated in the footpad with WNV. **a**, Immunofluorescence confocal microscopy imaging of sections of cerebral cortex at 5 dpi; Iba1 (white) and nuclei (Hoechst 33258, blue). Scale bar, 100 µm. Data are representative of 2 experiments, from left to right, with n = 6, 6, 8, 8, 8, and 8 mice per group. **b**, Relative Iba1-positive area was measured from at least 20 identified microglia/macrophage cells in each field and then divided by the number of microglia/macrophage nuclei counted. **c**, Total numbers of Iba1-positive microglia/macrophage in each field were counted. (**b-c**) Each data point reflects an average of the values obtained from at least 3 independent fields per mouse brain. Bars indicate mean values. Data are representative of 2

experiments, with from left to right, n = 6, 6, 8, 8, 8, and 8 mice per group. Statistical analysis: one-way ANOVA with Šídák's post-test, from left to right (****P < 0.0001, **P = 0.0016 (**b**); ***P = 0.0006, ns, not significant (**c**).

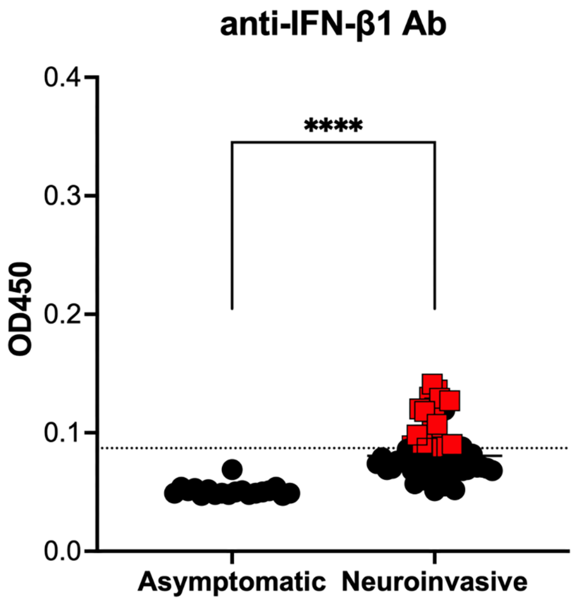

Supplementary Figure 9. Auto-Abs against IFN-β in WNV-infected human subjects. The optical density (O.D., 450 nm) values of anti-IFN-β1 auto-Abs in each WNV-infected cohort (asymptomatic, n = 56 and neuroinvasive, n = 19). Data is from 1 experiment performed in duplicate. Statistical analysis: two-tailed Mann–Whitney test: ****$p < 0.0001$.

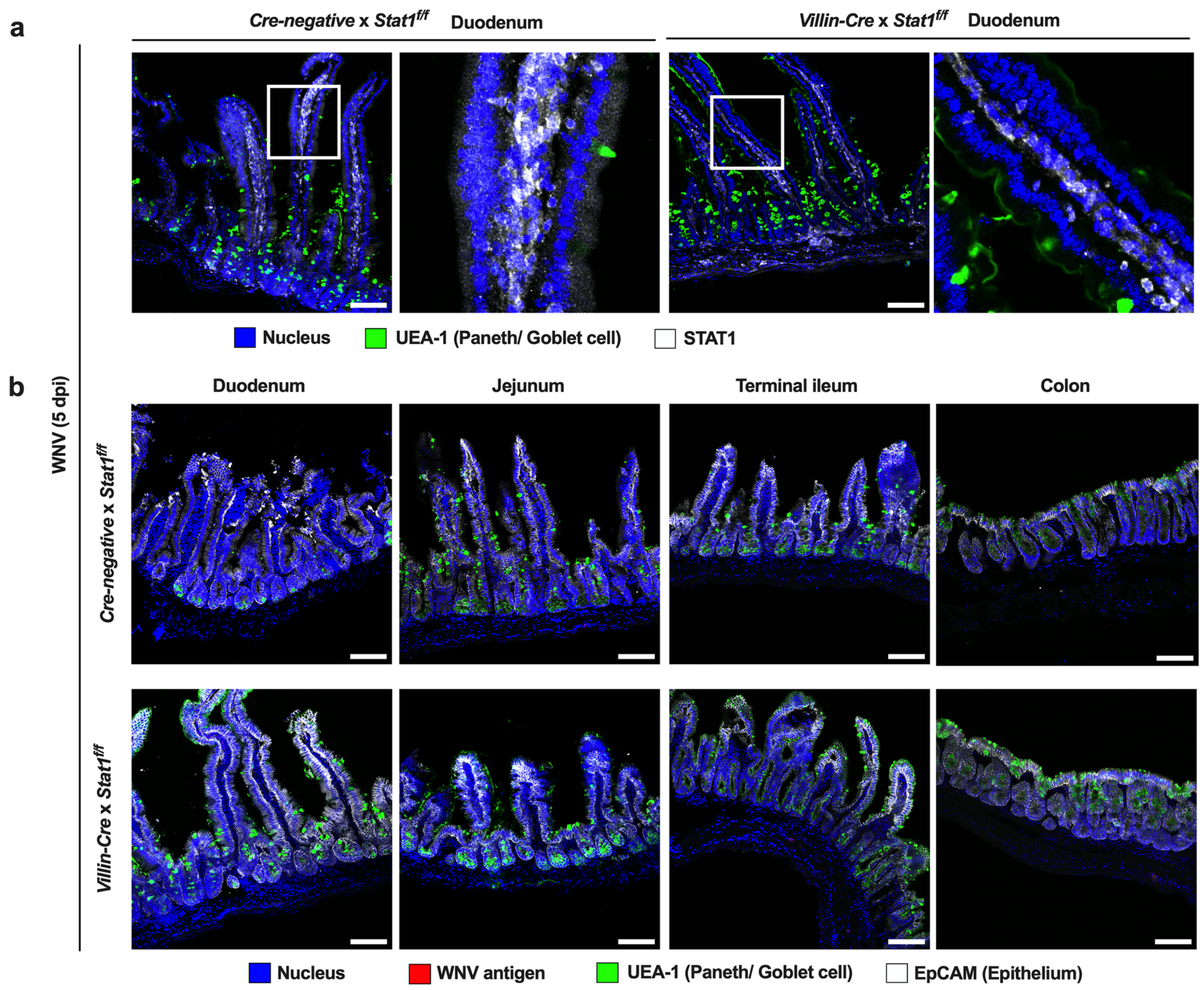

Supplementary Figure 10. Enterocytes in the GI tract of Villin-Cre *Stat1*$^{f/f}$ mice are not permissive to WNV infection. a-b, Cre-negative and Villin-Cre *Stat1*$^{f/f}$ SPF mice were inoculated subcutaneously in the footpad with 10^2 FFU of WNV, and sections of the GI tract were harvested at 5 dpi. Immunofluorescent confocal imaging of sections of WNV-infected *Villin-Cre x Stat1*$^{f/f}$ mice or littermate control Cre-negative mice (**a**) from the duodenum showing STAT1 (white), Paneth and goblet cells (UEA-1, green), and nuclei (Hoechst 33258, blue), and (**b**) along the GI tract showing WNV antigen (red), Paneth and goblet cells (UEA-1, green), EpCAM (white), and nuclei (Hoechst 33258, blue). Data are representative of 2 experiments with n = 8 mice per genotype. Scale bar, 100 μm.

Supplementary Table 1. Bacterial growth from serum and liver homogenates of WNV-infected SPF mice.

Condition	Infection	Number of animals tested	Dilution tested	Source	Results
Isotype	WNV, 5 dpi	5	Undiluted and 1:10 dilution	Blood	No bacterial growth
anti-IFNAR1	WNV, 5 dpi	10	Undiluted and 1:10 dilution	Blood	No bacterial growth
Isotype	WNV, 5 dpi	4	1:1 and 1:10 dilution	Liver	No bacterial growth
anti-IFNAR1	WNV, 5 dpi	6	1:1 and 1:10 dilution	Liver	No bacterial growth

Undiluted and diluted blood or liver homogenate samples were cultured on blood agar plates.
