# Supplementary material for: Blockade of interferon signaling decreases gut barrier integrity and promotes severe West Nile virus disease
Source: Nat Commun. 2023 Sep 25;14:5973. doi: 10.1038/s41467-023-41600-3 (PMC10520062; doi:10.1038/s41467-023-41600-3)
Supplement: Supplementary file 2 — Reporting Summary [file 41467_2023_41600_MOESM2_ESM.pdf]

nature portfolio

Corresponding author(s): Michael Diamond

Last updated by author(s): 08/22/2023

Reporting Summary

Nature Portfolio wishes to improve the reproducibility of the work that we publish. This form provides structure for consistency and transparency in reporting. For further information on Nature Portfolio policies, see our Editorial Policies and the Editorial Policy Checklist.

Statistics

For all statistical analyses, confirm that the following items are present in the figure legend, table legend, main text, or Methods section.

n/a	Confirmed	
☐	☒	The exact sample size (*n*) for each experimental group/condition, given as a discrete number and unit of measurement
☐	☒	A statement on whether measurements were taken from distinct samples or whether the same sample was measured repeatedly
☐	☒	The statistical test(s) used AND whether they are one- or two-sided<br>*Only common tests should be described solely by name; describe more complex techniques in the Methods section.*
☒	☐	A description of all covariates tested
☐	☒	A description of any assumptions or corrections, such as tests of normality and adjustment for multiple comparisons
☐	☒	A full description of the statistical parameters including central tendency (e.g. means) or other basic estimates (e.g. regression coefficient) AND variation (e.g. standard deviation) or associated estimates of uncertainty (e.g. confidence intervals)
☐	☒	For null hypothesis testing, the test statistic (e.g. *F*, *t*, *r*) with confidence intervals, effect sizes, degrees of freedom and *P* value noted<br>*Give P values as exact values whenever suitable.*
☒	☐	For Bayesian analysis, information on the choice of priors and Markov chain Monte Carlo settings
☒	☐	For hierarchical and complex designs, identification of the appropriate level for tests and full reporting of outcomes
☒	☐	Estimates of effect sizes (e.g. Cohen's *d*, Pearson's *r*), indicating how they were calculated

Our web collection on statistics for biologists contains articles on many of the points above.

Software and code

Policy information about availability of computer code

Data collection	No software was used in this study to collect data
Data analysis	Prism 8.0 was used to perform all data analysis. Confocal microscopy images images were acquired using a Zeiss Laser Scanning disk microscope 10x objective (NA 0.3), stitched (ZEN Blue software 3.7, Zeiss) and processed and analyzed by Fiji software (https://fiji.sc/Fiji).

For manuscripts utilizing custom algorithms or software that are central to the research but not yet described in published literature, software must be made available to editors and reviewers. We strongly encourage code deposition in a community repository (e.g. GitHub). See the Nature Portfolio guidelines for submitting code & software for further information.

Data

Policy information about availability of data

All manuscripts must include a data availability statement. This statement should provide the following information, where applicable:

- Accession codes, unique identifiers, or web links for publicly available datasets
- A description of any restrictions on data availability
- For clinical datasets or third party data, please ensure that the statement adheres to our policy

All data supporting the findings of this study are available within the main text, supplemental data, and source data.

Research involving human participants, their data, or biological material

Policy information about studies with human participants or human data. See also policy information about sex, gender (identity/presentation), and sexual orientation and race, ethnicity and racism.

Reporting on sex and gender	Sex data is provided in aggregate in Table 1. Gender data was not acquired. This information is not linked to individual disease manifestations or other identifiers.
Reporting on race, ethnicity, or other socially relevant groupings	Ethnicity/Race data is provided in aggregate in Table 1. This information is not linked to individual disease manifestations or other identifiers.
Population characteristics	Age is provided in aggregate. There were no prior underlying co-morbidities or diagnoses acquired or indicated.
Recruitment	Serum samples from asymptomatic WNV subjects identified through blood bank donations and a cohort of subjects with severe WNV neuroinvasive disease (Houston West Nile Cohort, Table 1) were obtained with written informed consent under approved protocols following the guidelines of the Human Investigations Committees of The University of Texas Health Science Center, Baylor College of Medicine, and Yale University School of Medicine, aliquoted, and then stored at -80oC . Severity of WNV infection was determined at the time of acute illness according to CDC guidelines (http://www.cdc.gov/ncidod/dvbid/westnile/clinicians/clindesc.htm). Asymptomatic, acutely infected subjects were identified via nucleic acid amplification testing by Gulf Coast Regional Blood Center, and an absence of illness history was confirmed by study coordinators. Clinical cases captured through public health surveillance were referred by the City of Houston Health Department and Harris County Public Health and Environmental Services to the study PI (Murray) for recruitment and enrollment.
Ethics oversight	The studies received Institutional Review Board approval from Baylor College of Medicine (H-30533).

Note that full information on the approval of the study protocol must also be provided in the manuscript.

Field-specific reporting

Please select the one below that is the best fit for your research. If you are not sure, read the appropriate sections before making your selection.

☒ Life sciences ☐ Behavioural & social sciences ☐ Ecological, evolutionary & environmental sciences

For a reference copy of the document with all sections, see nature.com/documents/nr-reporting-summary-flat.pdf

Life sciences study design

All studies must disclose on these points even when the disclosure is negative.

Sample size	No sample sizes were chosen a priori but were based on prior experiments and power calculation estimates. All experiments with statistical analysis were repeated at least two independent times, each with multiple technical replicates. Experimental size of animal cohorts was determined based on prior experience performing studies in mice.
Data exclusions	No data was excluded.
Replication	All experiments had multiple biological and/or technical replicates and are indicated the Figure legends.
Randomization	For animal studies, mice were randomly assigned to treatment groups in an age-matched distribution. For human subjects, the severity of WNV infection was determined at the time of acute illness according to CDC guidelines (http://www.cdc.gov/ncidod/dvbid/westnile/clinicians/clindesc.htm
Blinding	Experimental animals were randomized throughout the study, and scoring of immunofluorescence and histology images was performed in a blinded manner.

Reporting for specific materials, systems and methods

We require information from authors about some types of materials, experimental systems and methods used in many studies. Here, indicate whether each material, system or method listed is relevant to your study. If you are not sure if a list item applies to your research, read the appropriate section before selecting a response.

Materials & experimental systems

n/a	Involved in the study
[ ]	[x] Antibodies
[ ]	[x] Eukaryotic cell lines
[x]	[ ] Palaeontology and archaeology
[ ]	[x] Animals and other organisms
[ ]	[x] Clinical data
[x]	[ ] Dual use research of concern
[x]	[ ] Plants

Methods

n/a	Involved in the study
[x]	[ ] ChIP-seq
[x]	[ ] Flow cytometry
[x]	[ ] MRI-based neuroimaging

Antibodies

Antibodies used	Anti-IFNAR1 antibody (Leinco #I-401, clone MAR1-5A3), mouse IgG1 isotype control antibody (Leinco #I-117, clone HKSP84), anti-TNF-a antibody (Biolegend #506352, clone MP6-)T22), rat IgG1 isotype control antibody (Invitrogen, #14-4301-85), rat anti-WNV hyperimmune serum (Diamond laboratory), rabbit anti-EpCAM polyclonal serum (1:2000, Abcam #ab71916), rabbit anti-STAT1 monoclonal antibody (1:1000, Cell Signaling #14994), AF594-conjugated donkey anti-rat antibody (1:1000, ThermoFisher #A-21209), AF647-conjugated donkey anti-rabbit antibody (1:1000, ThermoFisher #A-31573), fluorescein-conjugated Ulex Europaeus Agglutinin I (UEA-1, 1:2000, ThermoFisher #L32476), Fc-specific goat anti-human IgG/IgA/IgM (1:10,000, Nordic Immunological Laboratories #GAHu/Ig(Fc))
Validation	All primary Abs were validated using purified viral proteins using an ELISA and confirmed to have the correct species and tareget specificity. All secondary antibodies were validated by the manufacturer per their associated DataSheets.

Eukaryotic cell lines

Policy information about cell lines and Sex and Gender in Research

Cell line source(s)	HIE cultures (J2 and J2 STAT1-KO) were previously described (see REF 86) and purchased (Digestive Disease Core, Baylor College of Medicine). Mouse enteroids were established from duodenum and colon tissues obtained from wild-type and Stat1-/- C57BL/6J mice (Precision Animal Models and Organoids Core, Washington University). BHK21-15 cells (C-13, CCL-10) and Vero cells (CCL-81) were obtained from the ATCC
Authentication	The KO enteroid lines were authenticated with antibody staining. All cell lines grew as expected with appropriate morphology and propagated virus as expected.
Mycoplasma contamination	All cell lines are routinely tested each month and were negative for mycoplasma.
Commonly misidentified lines (See ICLAC register)	This study did not involve any commonly misidentified cell lines.

Animals and other research organisms

Policy information about studies involving animals; ARRIVE guidelines recommended for reporting animal research, and Sex and Gender in Research

Laboratory animals	Wild-type C57BL/6J (Jackson Laboratories, #000664) were obtained commercially. Ifnar1-/- [B6(Cg)-Ifnar1tm1.2Ees/J, RRID: IMSR_JAX:028288, (74)], Ifngr-/- [B6.12957-Ifngatm1Agt/J, RRID: IMSR_JAX:003288, (75)]], Ifnlr1-/- [Ifnlr1tm1Palu, (76)], Stat1-/- [B6.129S(Cg)-Stat1tm1Dlv/J, RRID: IMSRJAX:012606, (77)], Villin-Cre [B6.Cg-Tg(Vil1-cre)997Gum/J, RRID: IMSR_JAX:004586 (78)] and Statlf/f [B6;1295-StatltmlMam/Mmjax, RRID: MMRRC_032054-JAX (79)] mice (all congenic on a C57BL/6 background) were bred under pathogen-free conditions at Washington University. C57BL/6J gnotobiotic mice were bred and housed at the Washington University Gnotobiotic Core Facility, and the GF status was confirmed through 16S qPCR analysis of fecal samples (Charles River). Mice were housed in groups of 3 to 5. Photoperiod = 12 hr on:12 hr off dark/light cycle. Ambient animal room temperature is 70° F, controlled within ±2° and room humidity is 50%, controlled within ±5%. All animals were used between 7 and 10 weeks of age.
Wild animals	No wild animals were used in this study.
Reporting on sex	While several of the studies shown were derived from male mice, the studies also were performed with both sexes.
Field-collected samples	No field collected samples were used in this study.
Ethics oversight	All experiments were conducted with approval of the Institutional Animal Care and Use Committee at the Washington University School of Medicine (Assurance number A3381-01)

Note that full information on the approval of the study protocol must also be provided in the manuscript.

Clinical data

Policy information about clinical studies

All manuscripts should comply with the ICMJE guidelines for publication of clinical research and a completed CONSORT checklist must be included with all submissions.

Clinical trial registration	N/A, not a clinical trial
Study protocol	Baylor College of Medicine, H-30533
Data collection	Data and specimens (serum) were longitudinally collected from enrolled human subject participants in Houston, TX between October 2002 and August 2022.
Outcomes	All participants had a confirmed infection with West Nile virus by PCR and/or IgM antibodies. Outcome measures were based on clinical presentation at the time of acute illness. If no clinical symptoms were present, they were classified as asymptomatic. If they were febrile and had other symptoms without neurological involvement, they were classified as West Nile fever. If they had evidence of meningeal involvement (pleocytosis in the CSF) in the absence of altered mental status, they were classified as West Nile meningitis. If they had pleocytosis plus altered mental status lasting 24 or more hours, then they were classified as West Nile encephalitis. Meningitis and encephalitis cases, with or without evidence of flaccid paralysis, were jointly classified as West Nile neuroinvasive disease cases.
